# Citizen science reveals temporally structured insect attraction and mitigation efficacy under artificial light at night

**DOI:** 10.1038/s42003-026-10979-2

**Published:** 2026-09-21

**Authors:** Johanna E. Reinhard, Manuel Dietenberger, Helene Rehberg, Rana Hofmann, Pia E. Lühr, Niklas Loubies von Harpe, Lena N. Vogel, Thea Wächter, Sibylle Schroer, Andreas Jechow, Franz Hölker

**Affiliations:** 1 Leibniz Institute of Freshwater Ecology and Inland Fisheries (IGB), Berlin, Germany; 2 Bertha-von-Suttner Gymnasium, Potsdam, Germany; 3 Department of Engineering, Brandenburg University of Applied Sciences, Brandenburg an der Havel, Germany; 4 Chair of Nature Conservation and Landscape Ecology, Albert-Ludwigs-Universität Freiburg, Freiburg, Germany; 5 Institute of Biology, Freie Universität Berlin, Berlin, Germany

**Keywords:** Conservation biology, Entomology

## Abstract

Artificial light at night (ALAN) is an anthropogenic disturbance with negative effects on nocturnal flying insects, yet most studies treat “night-time” as a uniform exposure period. However, nocturnal insects exhibit pronounced temporal niche partitioning, suggesting that attraction to artificial light and the effectiveness of mitigation measures may vary over the night. Using a citizen science framework, we show how insect attraction along a lakeside road varies across five nightly intervals, comparing conventional high pressure sodium with tailored and shielded LED road lights. Complementary UV ground traps showed marked shifts in local insect abundance and taxonomic composition, with peak attraction occurring during late evening and midnight hours (09.30pm-11.30pm-00.30am). Road lights closely followed this temporal trend. Tailored and shielded LED luminaires reduced total insect attraction, with the strongest effects during peak activity, but limited efficacy during twilight and late-night periods. Both total abundances and taxonomic composition showed significant time-by-treatment interactions, demonstrating that incorporating temporal dynamics can greatly improve targeted mitigation strategies.

## Introduction

### Artificial light at night (ALAN)

Global insect decline is a major environmental problem with implications for many important ecosystem services^1,2^. In this context, artificial light at night (ALAN) has emerged as a significant environmental stressor (light pollution), with wide-ranging effects on both terrestrial and freshwater ecosystems^3–5^, particularly for nocturnal species^6^. Besides being able to influence offspring developmental time and number^7^, ALAN interferes with many photoperiodic responses. This includes e.g. a regulation of diapause induction of moth species, resulting in miss-timed development and increased mortality^8,9^. ALAN further disrupts natural behaviours such as foraging, mating and migration across various taxa^10–14^. Moreover, nocturnal activity of insects may be higher than diurnal activity (with large differences between terrestrial and aquatic ecosystems), indicating that a large proportion of species interactions will be affected^11,15^. This may be particularly relevant in the context of the observed maladaptive attraction of many insect species to artificial light sources, such as road lights, which can have cascading effects on natural environments. Among the most affected insects in this context are highly seasonal aquatic taxa that complete part of their life cycle as flying adult stages, including *Ephemeroptera* (mayflies), *Nematocera* (midges) and *Trichoptera* (caddisflies)^16,17^. These insects play critical roles in aquatic ecosystems, particularly in terms of nutrient cycling, food web dynamics, and organic matter decomposition and bear many species vulnerable to anthropogenic stress^18,19^. *Ephemeroptera*, in particular, are known for their unique life cycle and emergence patterns, with nymphs spending most of their life underwater before emerging as adults to reproduce within a short time window^20,21^. However, adult flying *Ephemeroptera*, especially in urban and peri-urban areas and in the vicinity of waterbodies (as is the case at our experimental site), are at risk of being drawn to road lights and other sources of artificial light across habitat boundaries. This can lead to increased mortality and changes in overall population dynamics^17,22,23^. Similarly, *Nematocera* and *Trichoptera* larvae inhabit aquatic environments and adults represent important prey for a variety of species, from fish to birds^19,24^.

### Temporal niche partitioning

Timing is critical for a wide range of insects’ nocturnal behaviours, due to altered visual and thermal conditions of the night^14,15,25^. Numerous nocturnal species display thus temporal patterns of activity that are closely synchronized with the transitions of dusk and dawn. Many beetles (*Coleoptera*) are commonly most active soon after dusk^26–28^. Similarly, aquatic insects often synchronize their mass emergence or swarm activity to nocturnal hours^29^. These timing strategies not only reduce daytime exposure to predators^30^, but also track environmental cues, particularly humidity and temperature, that favour successful adult emergence and reproduction^31^. Consequently, nocturnal insect abundance is far from being temporally uniform throughout the night. Early evening often sees large swarms of *Nematocera* or the first emergences of *Ephemeroptera*^8,32,33^, whereas midnight to pre-dawn periods are frequently dominated by *Trichoptera* or by later waves of *Ephemeroptera* emergence^22,31^. This variation underscores that “night-time” comprises multiple ecological time windows partitioned by different insect taxa according to their evolutionary and physiological requirements. Since ALAN has been found to alter the timing and frequency of insect emergence^34^, which in turn disrupts predator-prey relationships and affects species that depend on these insects as a food source^35^, recognizing potentially taxon-specific temporal activity peaks enables researchers to assess ALAN induced impacts more precisely and to tailor mitigation strategies accordingly.

### Mitigation of ALAN

**Fig. 1:**
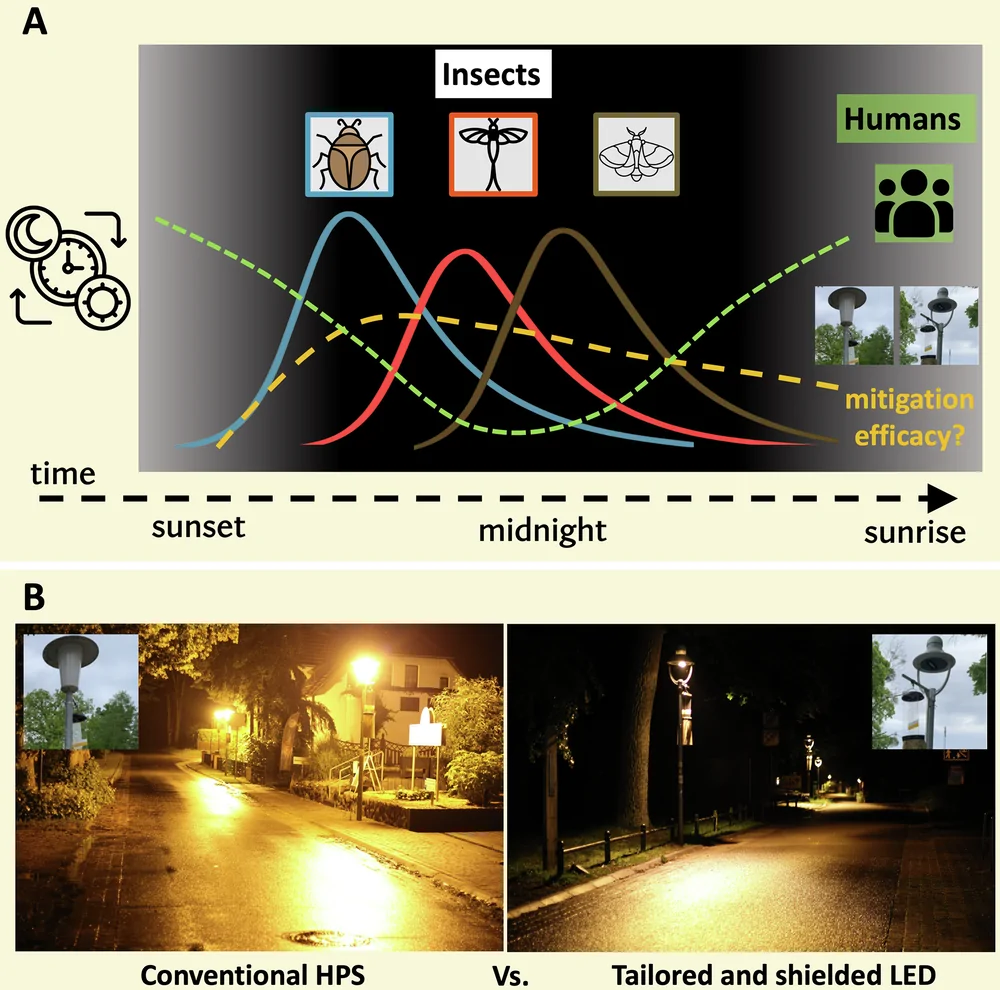
Conceptual framework of temporal structuring in ALAN attraction and mitigation efficacy under artificial light. **A** Schematic representation of temporally structured insect activity and attraction to ALAN across the night. Different insect taxa exhibit distinct activity peaks (solid curves), resulting in time-dependent attraction to ALAN. The effectiveness of mitigation measures (e.g. shielding, dimming, spatial tailoring; dashed yellow curve) is therefore not constant but varies across the night. Human activity patterns are shown for context (green dashed curve). Curves are illustrative and do not represent empirical data. **B** Comparison of road light treatments. Left: Conventional cylindrical high-pressure sodium (HPS) luminaires (2000K, 4.0 lx). Right: Tailored and shielded LED luminaires type “Selux BETA shield” (2200K, 4.1 lx). Figure created manually by AJ, icons used from Freepik package at flaticon.com, all photos by AJ).

ALAN can mask natural light patterns, thereby blurring temporal niches and potentially delaying or advancing behavioural events and disrupting established temporal partitioning^13,14,36^ by flattening the natural contrast between dusk, night, and dawn^6,37^. Given its growing recognition as an environmental pollutant, efforts to reduce ALAN’s impacts such as insect attraction through environmentally-friendly lighting solutions have gained increasing attention^6,38^. Although the exact flight-to-light behaviour is still a riddle and may be species-specific^39–41^, tailored and shielded LED road lights are one promising mitigation measure from an interspecific perspective that seems to outperform spectral tuning and dimming on many occasions^42^. In a previous study, we used tailored LED luminaires that direct light downward exclusively onto the target area using specialized optics. This minimizes light spill into the flight paths of insects and an additional shielding reduces the visibility of luminaire heads from a distance^43,44^. However, in that study the night was treated solely as one uniform exposure period. In the present study, conducted in Neuglobsow, a small village in Brandenburg (Germany), we compare conventional top-mounted cylindrical high-pressure sodium (HPS) luminaires with newly developed top-mounted LED luminaires with the same tailored and shielding technology but a traditional appearance (Fig. 1, bottom panels).

### Citizen science

One approach to conducting large-scale ecological studies with high sample sizes is through citizen science (CS). This is a research model that actively involves non-professional participants in data collection, monitoring and analysis. CS has been used successfully in a wide range of ecological and environmental research, allowing researchers to gather large amounts of data over vast geographical areas and time periods. Not only does CS provide an effective way to address challenges such as limited funding and time constraints, but it also engages the public and raises awareness about environmental issues^45–47^ including the night-time^48^. Specifically, involving school students in CS projects offers an opportunity to provide them with first-hand experience in scientific inquiry, data collection, and environmental research. It also contributes to their scientific literacy and fosters an appreciation for the natural world^49^. In this study^50^, high school students were involved as citizen scientists to study insect attraction to different lighting systems. Students examined the impact of road lights and ultraviolet radiation (UV) traps and co-designed an experimental protocol to analyse attraction patterns across different night-time intervals. Student participation was essential for collecting detailed night-time behavioural data over multiple years, providing a depth of observation that would have been difficult to achieve otherwise. By actively gathering data on insect attraction patterns at different times of the night, students played a pivotal role in the study’s success.

### Hypotheses

Despite growing evidence that insect activity is temporally structured, most ALAN studies implicitly treat night-time as one time frame. This overlooks the possibility that both insect attraction and the effectiveness of mitigation measures vary systematically over the course of the night. The conceptual framework underlying this time-resolved perspective is summarized in the top panel of Fig. 1. Here, we test this idea using temporally resolved samplings across five night-time intervals, combining citizen science with controlled comparisons of light sources (treatments): (a) cylindrical high-pressure sodium (HPS) road lights with conventional spatial light emission, (b) tailored and shielded LED road lights, and (c) UV ground traps. Due to their emission spectrum, the latter represent strong attractors to many nocturnal flying insects and were used to determine intervals of local peak insect attraction to ALAN^51,52^. Importantly, UV traps and road luminaires were evaluated independently. The UV data were used exclusively to characterise independent temporal variation in insect attraction across the night. This approach allowed temporal changes in abundance observed at road lights to be interpreted in the context of underlying attraction patterns, without conflating the two sampling methods. The night was divided into five time intervals: early evening (interval 1, 07.30 pm–09.30 pm), late evening (interval 2, 09.30 pm–11.30 pm), midnight (interval 3, 11.30 pm–01.30am), pre-dawn (interval 4, 01.30am–03.30am) and dawn (interval 5, 03.30am–06.00am).

We hypothesized that:

H1 Temporal attraction pattern: Light-mediated insect attraction is temporally structured across the night. We expected insect attraction (total abundances) at both UV ground traps and road lights to peak shortly after dusk to midnight, especially during intervals 2 and 3, and to decrease toward the late-night and dawn intervals^26,27^.

H2 Temporal mitigation efficacy of tailored and shielded road lights: Tailored and shielded LED road lights reduce insect attraction (total abundances) compared with conventional HPS road lights^43,44,53,54^. Because attraction is temporally structured, we expected the magnitude of this reduction ( = effect sizes) to be more pronounced during intervals of peak insect attraction, resulting in a significant interval-by-treatment interaction.

H3 Temporal and treatment-dependent order-level composition: The order-level composition of attracted taxa shifts over the course of the night (interval 1–5). At road lights, we expected this temporal compositional turnover to differ between conventional HPS and tailored and shielded LED luminaires, resulting in a significant interval-by.-treatment interaction in the multivariate analyses^27,43^.

## Results

### Effects of light treatments and time intervals

**Fig. 2:**
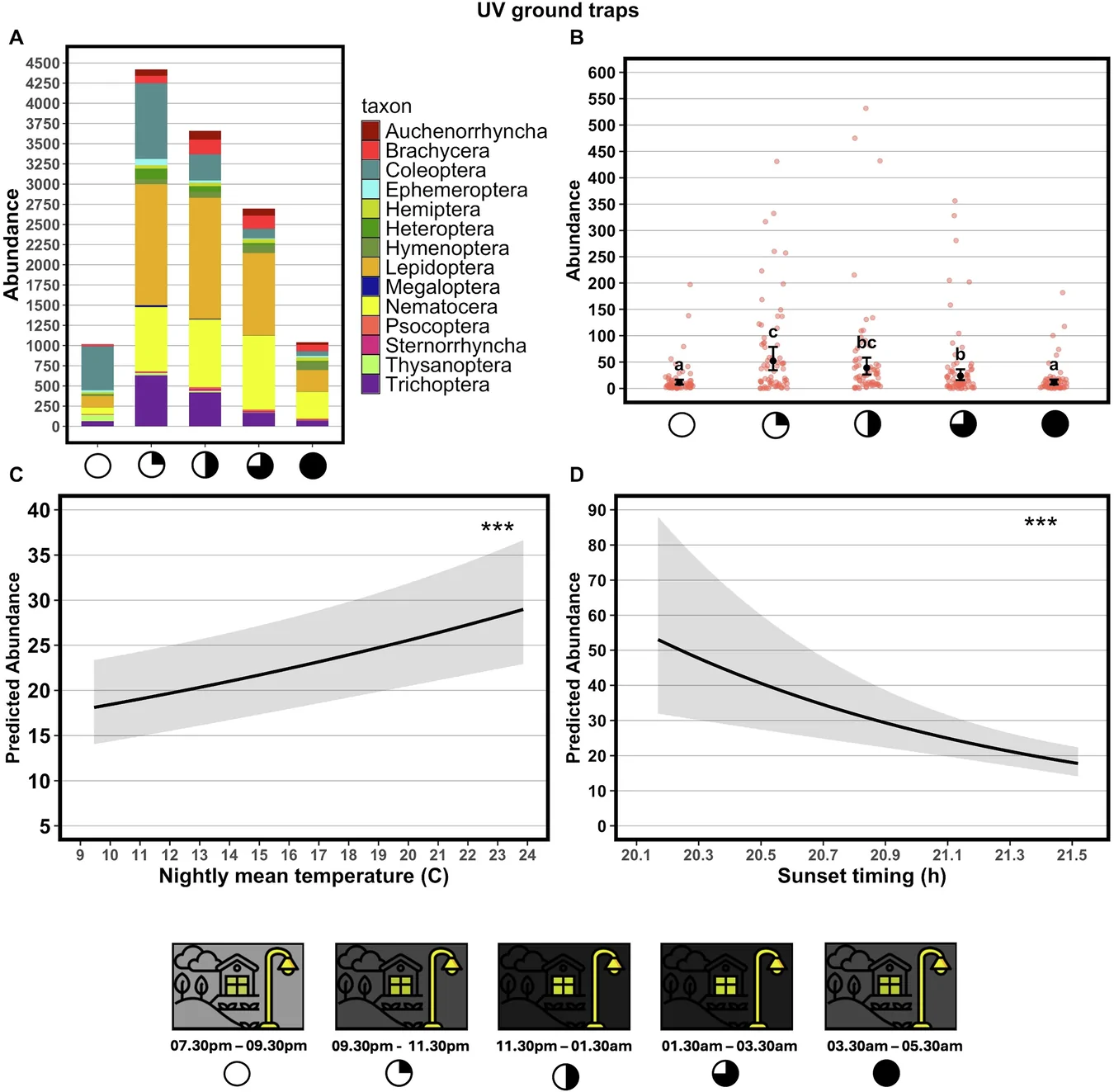
Time resolved abundances at ground UV traps. **A** Stacked total abundances per night-time interval, with colours indicating insect taxa. **B** Observed abundance per trap and sampling night across five night-time intervals (coloured points). Data for UV ground traps are based on *n* = 4 traps sampled over 19 nights between 2022 and 2024. Black points represent model-predicted abundances on the response scale (conditional means), and error bars the corresponding 95% confidence intervals (generalized linear mixed-effects model with negative binomial 2 error distribution). Compact letter displays refer to a pairwise post-hoc test between intervals (Tukey). Different letters indicate significant differences, whereas identical letters indicate no significant difference. **C** Nightly mean temperature and model-derived predicted abundance responses shown as an estimated marginal mean response curve, with shaded 95% confidence interval. **D** Sunset timing (h) and model-derived predicted abundance responses shown as an estimated marginal mean response curve, with shaded 95% confidence interval. Graphs created with R-Software Version 2024.04.2 + 764. Icons were modified with Microsoft® PowerPoint for Mac Version. 16.89.1 from Freepik package at flaticon.com.

A total of 12,828 insects were attracted to ground UV traps in 337 sampling events between 2022 and 2024 (Fig. 2, Supplementary Table S1). A total of 14 different insect taxa (orders) were observed at the ground UV traps. Most insects were attracted during intervals 2 (late evening, 34.5% of total) and 3 (midnight, 28.5%). Most *Coleoptera* were attracted in interval 2 (47% of total), *Ephemeroptera* similarly, with (48% of total). *Lepidoptera* exhibited two nearly equal peaks in intervals 2 and 3 (09.30 pm–11.30 pm–01.30am), corresponding to 34% of the total. In contrast, *Nematocera* displayed a later peak in interval 4 (pre-dawn, 31% of the total). Lower abundances were primarily observed in intervals 1 (early evening) and 5 (dawn) for most taxa.

**Fig. 3:**
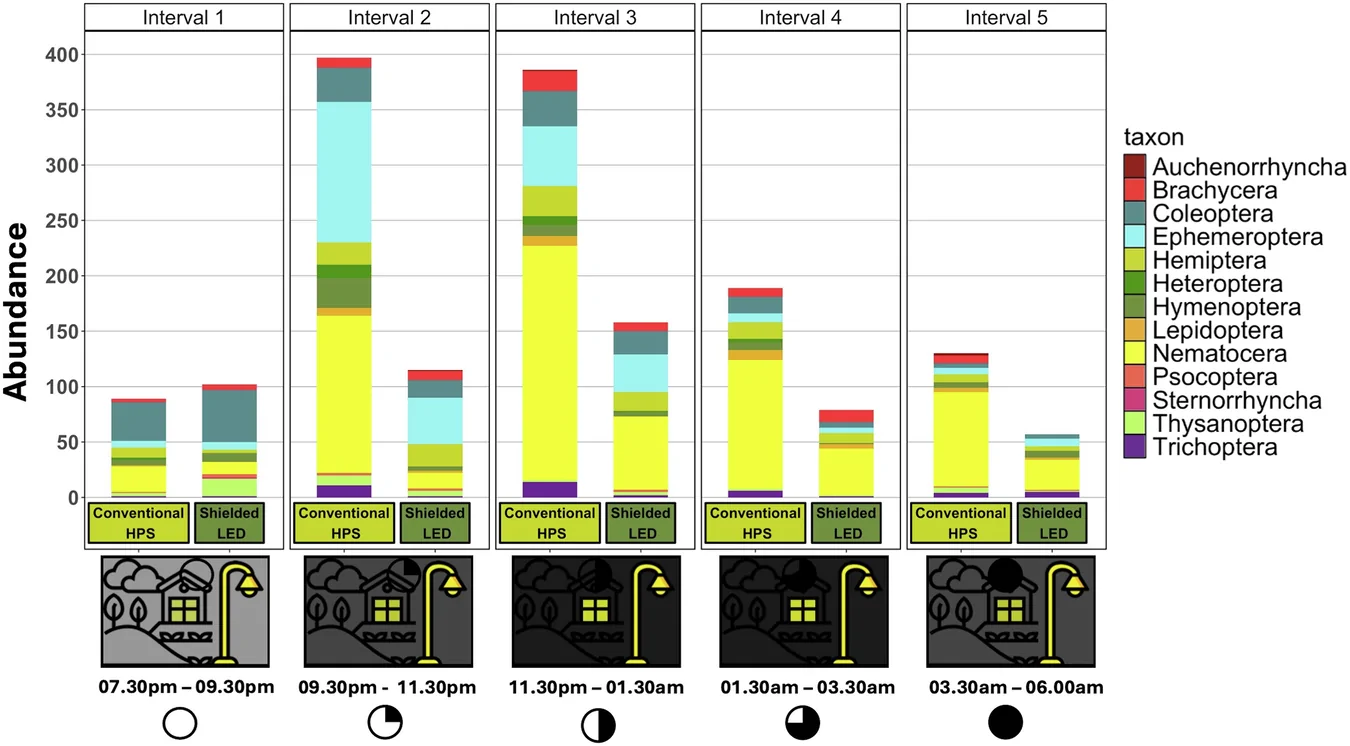
Time- and taxon-resolved insect abundances at road light traps per night-time interval. Bar plots show the total number of insects per taxon (colours) and treatment (HPS vs. tailored and shielded LED road lights) in different intervals (facets). Data for road light traps are based on *n* = 3 traps per treatment sampled over 13 nights between 2023 and 2024. Graphs created with R-Software Version 2024.04.2+764. Icons were modified with Microsoft® PowerPoint for Mac Version. 16.89.1 from Freepik package at flaticon.com.

A total of 1709 insects were attracted to flight-interception traps at road lights in 293 sampling events between 2023 and 2024 (1 trap per night per interval) (Fig. 3, Supplementary Table S2). A total of 13 different insect taxa (orders) were observed at these road lights. Again, most insects were attracted during intervals 2 (30.4% of total) and 3 (31.8% of total) (09.30 pm–11.30 pm–01.30am). Over twice as many insects were attracted to the conventional HPS road lights (1198 insects) compared to the tailored and shielded LEDs (511 insects). This represents a reduction by −57% at the novel luminaires. Similar reductions were observed for the most abundant taxa (*Nematocera*: −72%, *Ephemeroptera* −53%, *Coleoptera* −21%, *Lepidoptera* −73%). This effect was most pronounced during hours of peak attraction to road lights (e.g. interval 2: *Nematocera*: −90%, *Ephemeroptera* −67%, *Coleoptera* −48%, *Lepidoptera* −73%). A reduction in the number of different taxa (orders) under tailored and shielded luminaires was greatest in interval 2 (Supplementary Fig. S1).

A negative binomial regression showed that overall insect attraction at UV ground traps shifted over the course of the night (Fig. 2, Supplementary Table S3). Abundance increased significantly in intervals 2–4 relative to interval 1, with the strongest effect observed in interval 2. In contrast, abundance during interval 5 did not differ significantly from the baseline (interval 1). Nightly mean temperatures had a positive effect on insect attraction across time intervals, while sunset timing had a negative effect.

**Fig. 4:**
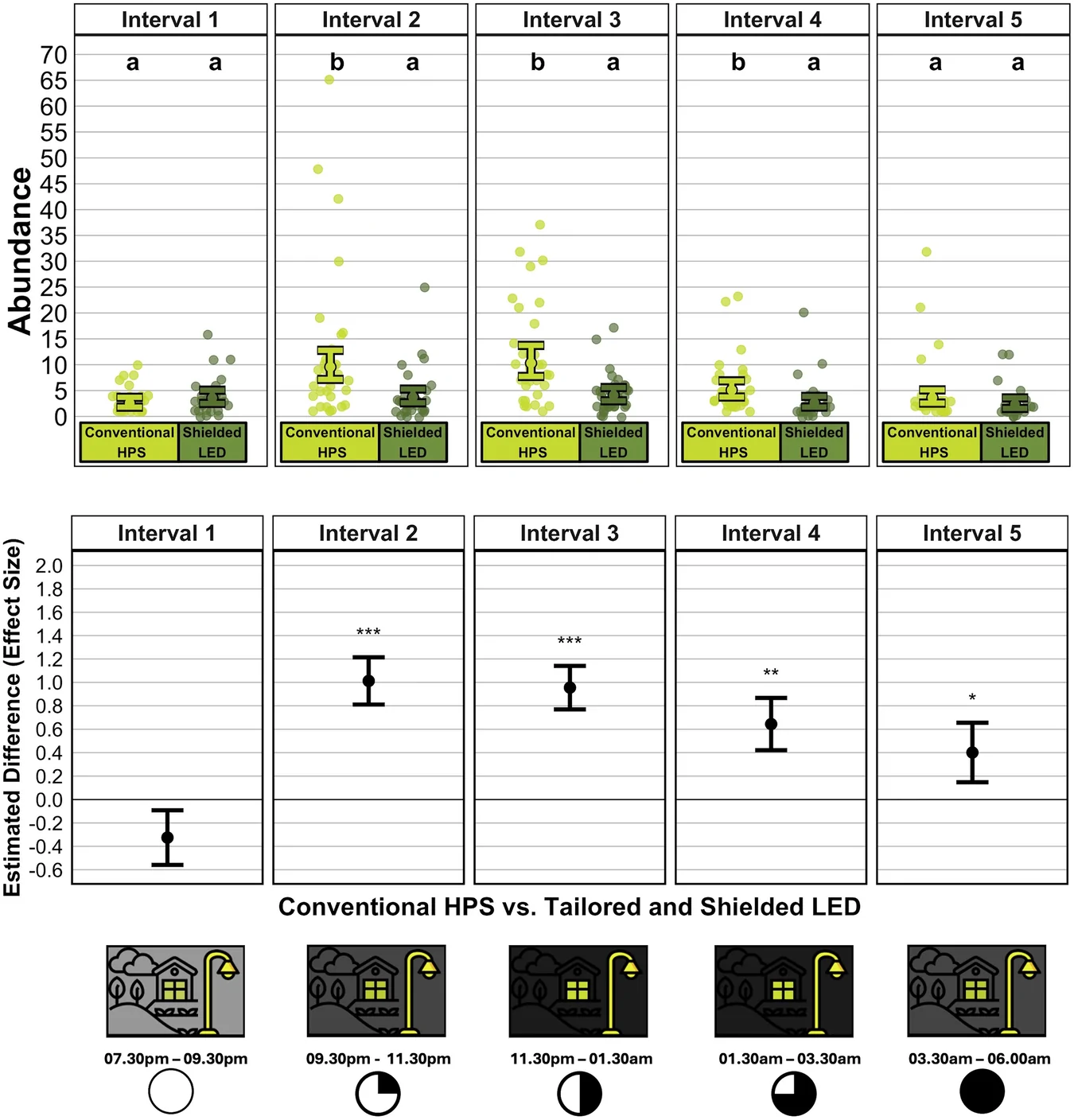
Generalized linear mixed model for the effects of light treatment and time interval at road lights. Top: Observed insect abundance per trap and sampling date (coloured points) in different intervals (facets) and for different light treatments (colours, HPS (yellow) vs. tailored and shielded LEDs (green)). Coloured points represent model-predicted abundances on the response scale (conditional means), and error bars the corresponding 95% confidence intervals (generalized linear mixed-effects model with negative binomial 2 error distribution). Compact letters refer to a pairwise post-hoc-test between treatments within each interval (Tukey). Different letters indicate significant differences, whereas identical letters mean no significant difference. Data for road light traps are based on *n* = 3 traps per treatment sampled over 13 nights between 2023 and 2024. Bottom: Model-derived estimated treatment differences (HPS vs. tailored and shielded LED) for each night-time interval, shown as contrasts of mean abundance (log scale) (points) with error bars representing ±1 standard error (SE) derived from the models estimated marginal means (generalized linear mixed model with negative binomial error 2 distribution). Graphs created with R-Software Version 2024.04.2 + 764. Icons were modified with Microsoft® PowerPoint for Mac Version. 16.89.1 from Freepik package at flaticon.com.

Insect attraction at road lights similarly peaked in intervals 2 and 3 (Fig. 4, Supplementary Table S4). Relative to interval 1, abundance significantly increased in interval 2–4, whereas interval 5 did not differ significantly from the earliest interval. The light treatment had a significant effect, with shielded and tailored roadlights predicting lower insect abundance across intervals 2–5. The interaction terms show that the reductions in insect abundance under shielded and tailored luminaires were absent in interval 1, became strongest during peak attraction in intervals 2 and 3 and remained significant, though less pronounced in interval 4 and 5. However, a subsequent direct pairwise comparison within intervals (Tukey posthoc test) showed that the treatment-difference in interval 5 was not significant. In contrast to the UV ground traps, nightly mean temperatures had a negative effect on insect attraction across time intervals and treatments, while sunset timing similarly negatively affected abundance.

### Composition of attracted taxa

**Fig. 5:**
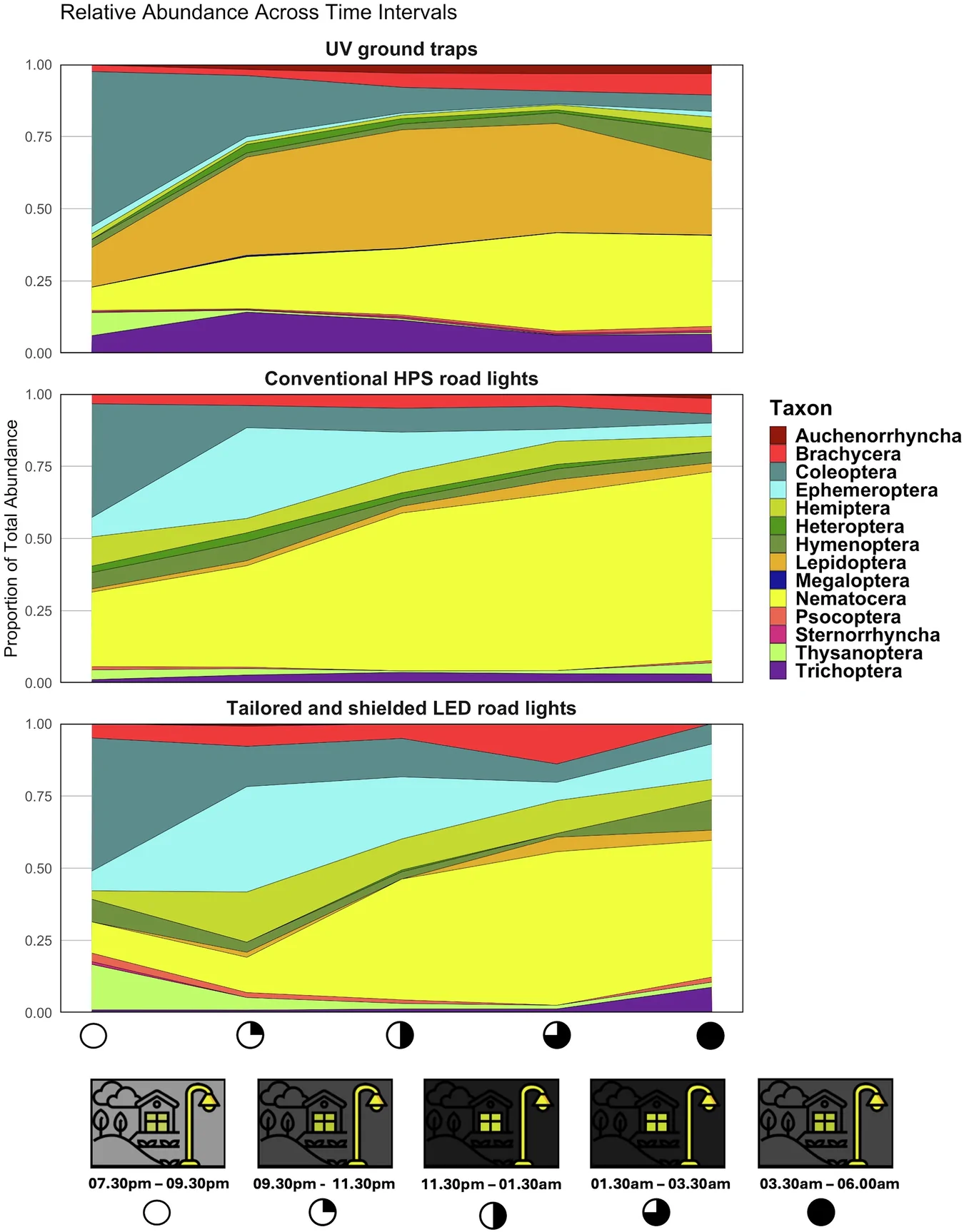
Time- and taxon-resolved relative abundance of insects across night-time intervals per treatment. Relative abundances of insects per taxon (colours) across five night-time intervals are shown for UV ground traps (top), conventional HPS road lights (middle), and tailored, shielded LED road lights (bottom). Data for road light traps are based on *n* = 3 traps per treatment sampled over 13 nights between 2023 and 2024, while UV ground traps are based on *n* = 4 traps sampled over 19 nights between 2022 and 2024. Graphs created with R-Software Version 2024.04.2 + 764. Icons were modified with Microsoft® PowerPoint for Mac Version. 16.89.1 from Freepik package at flaticon.com.

**Fig. 6:**
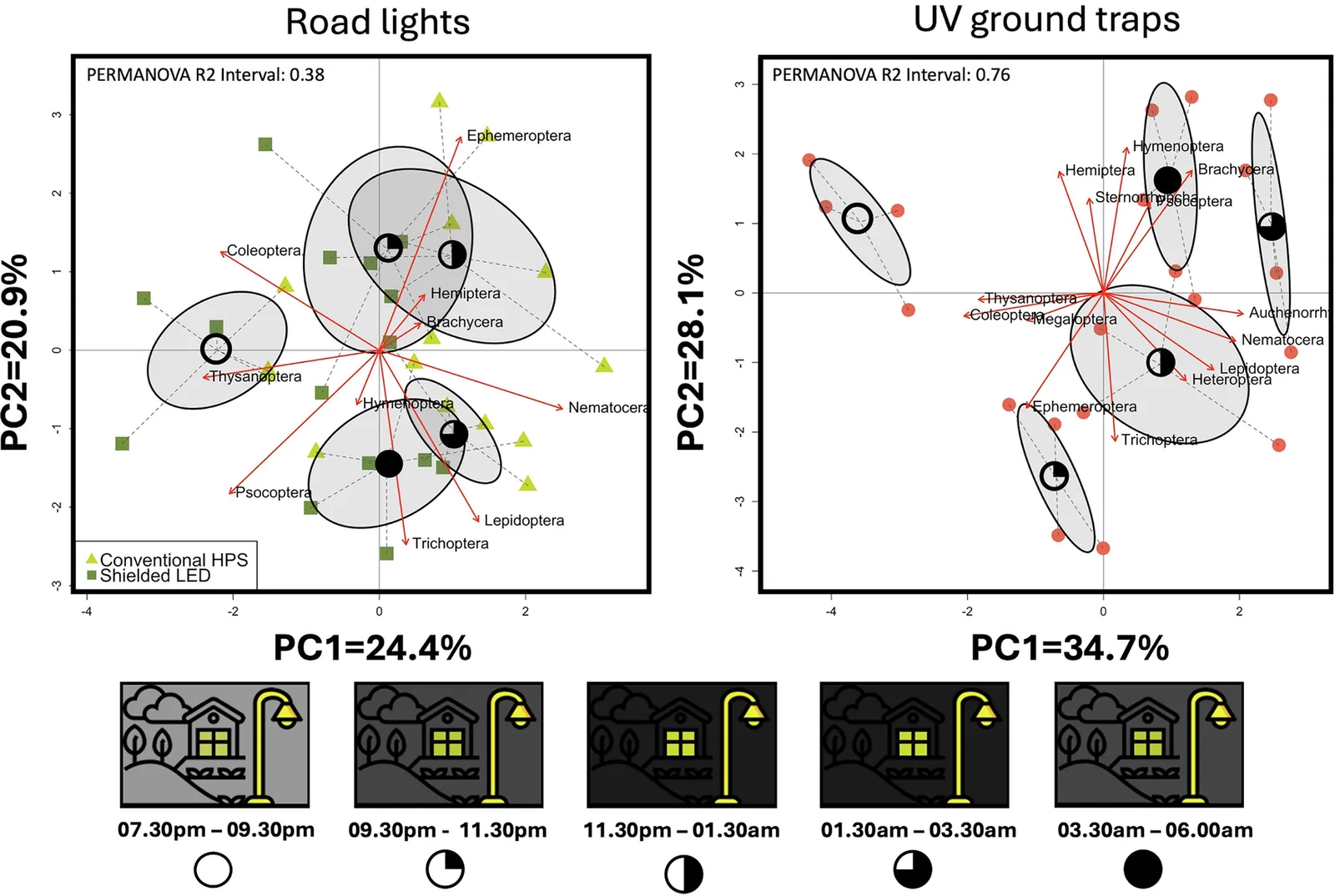
Principal component analysis (PCA) of insect community composition across night-time intervals. Left: Road lights with the two treatments: Conventional HPS vs. tailored and shielded LEDs. Each point represents an individual trap (*n* = 6) sampled across five night-time intervals, coloured by treatment. Right: UV ground traps. Each point represents an individual trap (*n* = 4) sampled across five night-time intervals. Ellipses indicate night-time interval groupings drawn at one standard deviation from the centroid of each interval group. Graphs created with R-Software Version 2024.04.2+764. Icons were modified with Microsoft® PowerPoint for Mac Version. 16.89.1 from Freepik package at flaticon.com.

To evaluate the influence of individual taxa on the multivariate variability between intervals and treatments (species composition) (Fig. 5, Supplementary Information Figure S2), we performed Principal Component Analysis (PCA) (Fig. 6).

PCA of UV ground traps (order-level abundances aggregated per trap and time interval) indicated that the first 4 principal components together explain 79.8% of the multivariate variance between traps in different treatments and intervals. Here, PC1 (34.7%) was strongest and negatively associated with *Coleoptera*, *Thysanoptera*. Highly abundant *Nematocera and Lepidoptera* and *Auchenorrhyncha* were positively associated. Further for UV traps, PC2 (28.1%) was negatively associated with *Trichoptera* and *Ephemeroptera* and positively with *Hymenoptera*, *Psocoptera, Sternorrhyncha, Hemiptera and Brachycera* (Fig. 6, Supplementary Table S5).

PCA of road lights (order-level abundances aggregated per trap, treatment and time interval) indicated that the first 4 principal components together explain 72.1% of the multivariate variance between traps in different treatments and intervals. For the road lights, PC1 (24.4%) was strongest and negatively associated with *Coleoptera*, *Thysanoptera and Psocoptera. Nematocera were strongly* positively associated. PC2 (20.9%) of road lights was negatively associated with *Lepidoptera* and *Psocoptera* and *Trichoptera* positively with *Ephemeroptera* (Fig. 6, Supplementary Table S6). Due to >70% zeros, 3 taxa (*Auchenorrhyncha, Heteroptera, Sternorrhyncha*) were excluded.

To further include a statistical measure, PERMANOVA based on Bray-Curtis dissimilarities demonstrated that both sampling interval and lighting treatment had significant effects on community composition at road lights (Supplementary Table S7). Community assemblages varied strongly across intervals (R² = 0.38, *p* = 0.001), indicating pronounced temporal changes. Lighting treatment also significantly influenced assemblage structure (R² = 0.13, *p* = 0.001), with distinct community compositions observed under different roadlight types (conventional HPS vs. tailored and shielded LED). Furthermore, a significant interaction between interval and treatment (R² = 0.13, *p* = 0.023) revealed that temporal patterns in community composition were not consistent across treatments, suggesting that the influence of road lighting type on assemblage structure depended on the time. Overall, the model explained 64% of the total multivariate variation in community composition.

PERMANOVA of UV traps similarly revealed a significant temporal variation in the species composition (R² = 0.76, *p* = 0.001) (Supplementary Table S8).

## Discussion

### Intervals

Temporally resolved sampling of five night time intervals gave valuable insights into insect attraction dynamics to ALAN. In this context, the UV traps (or “light traps”) on the ground were intentionally unshielded to maximize captures by exploiting many nocturnal insects’ strong short-wavelength sensitivity^8,55^ and were used as a parallel measure of relative light-mediated attraction under standardized conditions rather than a true experimental control (unlit). Therefore, the most insects (individuals) in this study were attracted to the UV traps. Especially moths have been observed to be attracted to UV- and blue wavelengths^51^. Conclusively, the relative abundance of *Lepidoptera* at UV traps (34,5%) was substantially higher than at the road lights (2,2%). As expected, this constitutes the largest difference between UV and road light traps. Importantly, *Lepidoptera* at UV traps remained active during intervals 2–5. However, overall insect abundance at UV traps peaked in the late-evening and midnight intervals 2 and 3 (consistent with H1). Road lights showed a similar temporal attraction pattern, indicating that sampling on ground level (UV traps) and higher up at the flight-interception traps (road lights) both reflected a local light-sensitive pool of insect species and a consistent temporal abundance pattern throughout the night, despite the differences in spectral composition and spatial distribution between UV traps and road lights.

Seasonal dynamics likely played an important role in shaping nocturnal insect attraction in this freshwater context study, particularly for taxa with short and synchronised adult phases such as *Ephemeroptera*^29,32^. However, our study did not aim at resolving specific responses at this temporal resolution and therefore does not extend beyond the three-month sampling period (June to August). Addressing these questions would require longer-term studies at multiple study sites and explicit integration of phenological and environmental drivers (e.g. temperature, humidity, wind speed, precipitation, moon elevation).

Despite the site-specific nature of our study, we are confident that our results reflect a general pattern of peak insect attraction shortly after sunset, particularly consistent with observations of Hao et al. and Charvalakis (2026)^27,28^. A combined assessment of overall temporal attraction patterns (total insect abundances), the composition of attracted insect orders and the mitigation efficacy of road light design under real operational lighting conditions remains, however, underexplored in light pollution research. In this context, the study by Charvalakis et al. in a forest context addresses this question from a complementary perspective, demonstrating that the timing of insect attraction was both taxon- and spectrum-dependent^28^. Similar to our study, overall phototactic attraction intensified from the early to the middle of the night but varied with taxon (order). However, the absence of a significant taxon-light colour interaction and the of lack of improvement in model fit (AIC) in their cumulative timing models indicates that spectral effects on the “centre of activity” were rather consistent across insect orders, even though additive models showed overall differences among insect orders. In addition, spectral effects were derived from post-hoc tests of order-specific models and not consistently present.

Similarly, Hao et al. do not explicitly test an interaction effect between spectrum and timing^27^. Nevertheless, although they used different trapping techniques (sticky boards) and light sources (mainly narrow-spectrum LEDs), they reported a similar temporal signature and observed that most insects were attracted to LEDs between 08:00 pm and 09.30 pm, ~1.5–3 h after sunset, before declining thereafter^27^. Notably, their study was also conducted in a suburban environment. The observed pronounced temporal peaks in overall abundance and importantly the temporal shift in taxonomic composition in our suburban study similarly indicate a temporal niche partitioning, reflecting taxon-specific circadian rhythms.

In contrast to existing research, our combined univariate and multivariate analyses reveal a significant interaction between timing and treatment (road lights), both in total insect abundances (H2) and composition of attracted taxa (orders) (consistent with H3), demonstrating that light treatment effects varied over time and across taxa while avoiding the need for separate order-specific models. These multivariate patterns are based on community matrices of order-level abundances, with compositional differences quantified using Bray-Curtis dissimilarities and tested via PERMANOVA. PCA provided a complementary unsupervised ordination, illustrating how specific taxa contribute to these temporal shifts in community structure. Notably, temporal intervals explained more variation in community structure than the lighting treatment at road lights. This temporally dynamic composition likely reflects differences in the diel activity patterns of insect taxa and was evident in both the PERMANOVA results and the PCA, where samples (individual luminaires) associated with UV ground traps and both road light treatments primarily separated along temporal gradients in taxonomic composition.

A finer taxonomic resolution than the order level used here could still show additional ecological patterns, including within-order shifts not detectable at our level of aggregation, and clarify whether specific functional groups, guilds or species benefit disproportionally. However, no insect order was more strongly attracted to the tailored and shielded LED roadlights than to conventional HPS roadlights (except very low counts of *Psocoptera* and *Sternorryncha*). Consistent with the PCA, which showed that taxonomic separation between treatments was secondary to temporal gradients in order-level community composition at road lights, we consider it unlikely that finer taxonomic resolution would alter the direction of the observed effects or reveal pronounced trade-offs at different times of the night. This is especially supported by the species-level results of Dietenberger et al., who demonstrated that tailored and shielded luminaires reduced not only abundance, but also the species richness of parasitoid wasps as bioindicator group^44^. Unlike spectral tuning approaches that are naturally related to species-specific preferences, tailoring and shielding primarily reduces overall light emissions, source visibility and subsequently the spatial extent over which behavioural response thresholds are exceeded. Mitigation effects are therefore expected to be less dependent on taxon-specific sensitivities (see also Owens et al.^42^) .

Moreover, nocturnal variation in abiotic conditions may have contributed to the observed temporal patterns in insect attraction. Sampling periods were chosen to coincide with the waxing moon phase to ensure a consistent influence of natural and artificial light on insect behaviour. However, in particular, declining air temperature over the course of the night may influenced the results. Although hourly temperatures were not measured on site, we show that nightly mean temperatures (DWD data) acted alongside intrinsic circadian rhythms in shaping the observed shifts in abundance, importantly across time intervals^25^.

The contrasting temperature effects observed between the UV ground traps and road lights may reflect differences in how insects respond to different light sources and sampling contexts. In the UV trap model, the positive relationship between temperature and insect abundance is consistent with a well-established increase in nocturnal insect activity at warmer temperatures^43^.

The UV ground traps were designed to maximize capture efficiency, such that increased activity on warm nights likely translated into higher trap counts. In contrast, the road light model showed a negative temperature coefficient, suggesting that insect attraction to road lights did not scale with temperature in the same way.

This discrepancy may be related to the observed differences in taxonomic composition, behavioural responses to light spectra or environmental conditions potentially correlated with temperature, such as humidity or wind speed. However, because temperature significantly improved model fit and represents an important ecological driver of insect activity, it was retained in the road light model to account for environmentally-driven variation and to reduce confounding of the interval-by-treatment effects. In this context, Fetnassi et al. demonstrated that abiotic variables can strongly influence insect trap catches and emphasized that different trapping methods may respond differently to environmental conditions because they rely on distinct behavioural cues^56^. While upward directed UV ground traps maximized attraction of phototactic insects, road lights may have altered insect movement and aggregation differently in this study. This further supported the inclusion of temperature as a covariate in our analyses to account for environmentally-driven variation.

Sunset timing, which acted as a proxy for varying photoperiodic parameters (e.g. night length, moon elevation) rather than exact photoperiod, had a negative effect on total insect attraction in our fixed time intervals. In other words, during shorter nights, fewer insects were attracted to UV ground traps and road lights. A possible explanation for this pattern is that intervals 1 and 5 increasingly overlapped with pre-sunset and dawn conditions during shorter nights, when reduced light contrast may have suppressed attraction. Reduced light contrasts under brighter ambient conditions may therefore have contributed to the observed temporal patterns in attraction. The inclusion of sunset timing as a fixed effect controlled for some night-to-night variation in insect attraction associated with changing ambient light conditions, but did not allow to fully separate this effect from genuine temporal variation in insect activity. However, we did not test for an interaction between time intervals and sunset timing. By modelling the interaction between time intervals and road light treatments along nightly mean temperatures, sunset timing and a random intercept for the year, our analyses accounted for temporal structure in insect responses among treatments while accounting for night-to-night variability, which always reflect causal underlying environmental gradients^43^.

Specifically, during the early evening (interval 1), the relative amount of *Coleoptera* (and *Thysanoptera*) strongly increased compared to later periods at road lights and UV traps. A similar attraction pattern during and shortly after sunset has been shown by other studies^27,28,57^, but may also depend on habitat type, region and abiotic factors^25^.

This shifted toward a greater dominance of Lepidoptera (especially at UV traps) and a larger portion of aquatic *Ephemeroptera* (especially at road lights, located closer to the neighbouring freshwater lake) during the subsequent evening (intervals 2 and 3). These attraction patterns align with research indicating structured, non-uniform activity patterns in aquatic insects. E.g. mayflies (*Ephemeroptera*) are expected to show peak activity during the early evening^32^ or late at night^22^ to optimize mating and egg-laying.

Furthermore, during midnight, the relative portion of highly abundant *Nematocera* became dominant at the road lights (intervals 3–5). In our study, the overall high abundance and proportion of *Nematocera* in intervals 3–5 indicated that *Nematocera* remained active during the whole night, which is consistent with the observations of Hao et al.^27^. Alternatively, a longer remaining time under the illumination and increased disorientation of *Nematocera* and consequently high capture rates played a role here^58^. Caglar and Alten documented species-specific peaks in mosquitoes (*Culicidae*): *Culex pipiens* and *Cochlerotatus dorsalis* activity was fairly evenly spread over the whole night, while *C. tritaeniorhynchus* exhibited bimodal peaks at 08:00 pm–10:00 pm and again at 12:00 pm–02:00am^33^.

Caddisflies (*Trichoptera*) tend to emerge closer to midnight or pre-dawn to minimize predation risk^31^. Also Brakel et al. observed that *Trichoptera* peaked between 10:30 pm and 11:00 pm, followed by a decline until approximately 02:00am–02:30am, with a minor resurgence before dawn. We observed a similar rebound of *Trichoptera* before dawn at tailored and shielded luminaires (interval 5). However, at UV ground traps, the overall higher *Trichoptera* abundance (compared to road lights) reached a proportional peak during interval 2. This was true despite a larger distance of the UV traps to the nearby freshwater lake compared to the road lights^59^, indicating that UV-intensive and upward directed ground traps were generally more attractive to *Trichoptera* than HPS or LED road lights in our study. In contrast, aquatic *Ephemeroptera* and *Nematocera* were predominantly attracted to road lights. This divergence may reflect taxon-specific differences in spectral sensitivity, orientation behaviour or dispersal capacity^59^.

In general, Kasai and Hironaka identified three broad patterns: one in which insects remain active from sunset until around 02:00am, another where activity extends until dawn (06:00am), and a smaller subset that persists into the morning (10:00am)^58^. The observed patterns in our study, while not assigning specific groups, similarly suggest that aquatic insect taxa partition night-time hours. Conventional HPS road lights, tailored and shielded LED road lights and UV traps showed this temporal partitioning in terms of overall abundance and importantly the composition of attracted taxa, which gave valuable insights into attraction dynamics that should be considered in light installations. For example we show that aquatic *Ephemeroptera* were drawn to the road lights, especially during interval 2 (09.00 pm–11.30 pm) and most likely from the nearby lake at the study site. This suggests that especially in these aquatic-to-terrestrial transition zones, local insect communities can profit from time-sensitive and mindful light planning when converting to LEDs (next section).

### Road lights

Tailored and shielded LED road lights (CCT = 2200K, 4.1 lx) attracted only 42.6% as many insects as conventional HPS (CCT = 2000K, 4.0 lx) road lights (consistent with H2), with a significant interaction between time interval and light type. Across all intervals, insect abundance was highest during intervals 2 and 3 (09:30am–11.30 pm–01:30am), with both intervals showing highly significant positive effects relative to the baseline (interval 1). Abundance declined thereafter, with a moderate increase during interval 4 (01.30am–03.30am) and no significant difference in interval 5 (03.30am–06.00am). The interaction term indicated that the effect of lighting type depended on the time of night. Specifically, insect abundance at tailored and shielded LEDs was significantly lower during intervals 2–5 with the strongest reductions observed during intervals 2 and 3 (interaction estimates = −1.33 and −1.28, *p* < 0.001). These estimates correspond to ~70% decrease in expected insect counts compared to HPS lights during peak attraction periods (on the response scale).

Although CCT was matched between treatments, this metric does not capture differences in spectral power distribution or insect visual perception. However, luminaires were standardised as far as possible under real-world conditions, with comparable light intensity and CCT. The primary distinction among treatments was therefore the light distribution, reflecting differences in light emission and shielding, source visibility respectively. While the broader spectral output of LEDs may increase their physiological relevance for insects compared to HPS (compare individual spectra Supplementary Fig. S5), tailored and shielded LED luminaires nevertheless attracted fewer insects despite this broader emission spectrum. This indicates that reductions in insect attraction were primarily driven by luminaire design rather than spectral composition.

The results confirm previous results that a transition from conventional luminaires to tailored and shielded LEDs reduces insect attraction across many taxa^43,44^. This is also supported by a recent study of Seymoure et al. They tested different “levels” of shielding in a dark desert environment and found that glare, more than uplight, attracted flying insects^60^. Notably, the tailored and shielded luminaire configuration tested here (Selux BETA shield top attachment) effectively reduced both uplight and glare, lowering the overall attraction of nocturnal flying insects, importantly under real-world application in a suburban environment. This may be especially relevant regarding a potentially reduced flight-to-light-behaviour under moderate skyglow and local competing light sources (as suggested for urban populations^61–63^).

The findings therefore emphasize the value of a tailored spatial light distribution and additional shielding to reduce flying insect attraction when retrofitting infrastructure in future municipal light planning. Beyond previous findings, the time resolved samplings conducted in this study show that this positive effect is most pronounced during hours of peak attraction approximately soon after sunset, as expected (H2). In our study, conducted from June to August, this corresponded to 09.30 pm–01.30am and a mean sunset time at 09.12 pm (min 20.10 pm, max 09.36 pm) during the sampling period.

The PCA and PERMANOVA analyses further revealed that both time interval and light treatment significantly shaped the composition of attracted taxa at road lights. Notably, temporal variation explained a large proportion of the observed multivariate variance, indicating that community assemblages shift markedly over the course of the night. Lighting treatment also exerted a clear influence on assemblage structure, though its effect size was smaller than that of time. However, the interaction between interval and treatment underscores that the influence of lighting technology on insect communities was not static but varies throughout the night (H2 and H3). In other words, the same luminaire type attracted different insect assemblages at different times, emphasizing the need for temporally explicit assessments in ecological lighting studies.

Overall, these results go beyond greater detectability during hours of peak attraction (total insect abundances). The multivariate analyses additionally show that observed total abundances varied in their taxonomic composition, depending on the time of the night. This underpins that, rather than simply reflecting differences in variance or sample size, this is unlikely to represent a statistical artefact. The significant interaction estimates therefore indicate that effect sizes themselves varied systematically across the night.

These observations extend our previous research on a higher temporal resolution and reinforce that emission geometry is a primary determinant of insect attraction to road lights^43^, importantly under suburban real-world application, which should be further tested across a wider range of contemporary luminaire designs. In general, the greatest gains from mitigation efforts are likely to be achieved during hours of overall peak attraction. While a conversion to tailored and shielded LED luminaires is considered a best practice standard, this is a long-term solution that naturally requires installations costs. Although not investigated in this study, “dimming” of highly controllable LED technology may constitute a functional alternative during hours of peak attraction where retrofitting is not possible. For example, Bolliger et al. showed that dimming luminaires during hours of low-traffic reduced insect attraction. Considering the gained temporal insights in this study, this could e.g. be tailored to sunset hours, especially relevant in protected areas with high *Coleoptera* diversity. In vicinity to waterbodies, this may be similarly relevant for *Ephemeroptera* during post-sunset hours and *Nematocera* during later periods^53,64^.

In summary, our results indicate that ALAN does not exert a uniform pressure on nocturnal insect communities, but interacts with temporally structured attraction patterns. Consequently, mitigation measures such as shielding, spatial tailoring, spectral tuning or dimming cannot be evaluated independently of time. Incorporating temporal resolution into ALAN research and lighting design may therefore substantially improve biodiversity outcomes without compromising human lighting needs.

### Citizen science

This study represents rare peer-reviewed ALAN/insect research conducted by school student citizen scientists in Germany. Their contributions on sustained sampling over multiple years were invaluable in capturing long-term behaviour patterns. A broad survey of 35 night-time CS projects confirms that volunteer programs operating under clear protocols can deliver data of professional quality while deepening public engagement with nocturnal ecology^48^. The students’ effort, recognized with several awards, therefore illustrates the dual dividends of citizen science: advancing ecological knowledge and fostering the next generation of researchers^64^. Reversing the steep decline in insect populations demands that monitoring and scientific practice become mainstream and that the gap between science and society is dismantled^1^. Involving students in research is a powerful way to bridge this divide, strengthening biodiversity monitoring, forecasting, protection and the bottom-up implementation of conservation policies^65^.

### Conclusion

This study revealed pronounced temporal dynamics in insect attraction to ALAN, with distinct taxa dominating at different night-time intervals. Such structured attraction patterns highlight that light-induced impacts on insects are not static but vary strongly over the course of the night. Importantly, tailored and shielded LED road lights (BETA shield top attachment) attracted less than half as many insects as cylindrical conventional HPS luminaires, demonstrating that emission geometry and shielding effectively reduce ecological disruption. Reductions in attraction occurred across multiple taxa and were accompanied by shifts in community composition relative to HPS lighting. However, temporal attraction patterns remained consistent between both treatments, indicating that tailored and shielded LED luminaires reduced attraction without altering nocturnal activity dynamics. Mitigation measures were most effective during periods of overall peak attraction to ALAN. By integrating these temporal insights, future lighting technology could integrate for example time-dependent spectral tuning or dimming of luminaires to further limit ecological consequences. The findings thus provide a practical framework for time-sensitive, biodiversity-friendlier lighting design.

## Methods

### Experimental design

The study was conducted in Neuglobsow, Brandenburg (Germany) near a freshwater lake: Lake Dagow (Supplementary Fig. S3). To investigate nocturnal insect attraction in response to ALAN, UV light traps (*n* = 4) and a non-randomized linear array of road lights with conventional cylinder HPS (CCT 2000K) column luminaires (*n* = 3) and tailored and shielded LEDs (Selux, BETA shield, CCT 2200 K) luminaires (*n* = 3) were monitored (Supplementary Table S9, Supplementary Figs. S3, S4). The distance between luminaires was ~30 m. The illuminance was almost identical for both road light treatments (4.0 lx for HPS and 4.1 lx for LEDs). For individual spectra of road lights see Supplementary Fig. S5. These novel road lights were developed to reduce spill lights in the trajectory of insects beyond the target area^43,50^.

Flight interception traps (*n* = 6) were used to monitor the attraction effect of road lights. Collection containers contained ~300–400 ml of 80% ethanol. Portable UV traps sometimes also called “light traps”, were positioned on the ground and were not visible from the road lights, ensuring independent results. The traps consist of a light source with three acrylic vanes in a funnel that rests in a 27 L container with egg cartons inserted in it. As a light source, a 50-cm-long 2835 SMD-LED-strip was used with peak emissions at a wavelength of about 400 nm wrapped around a 21 cm PVC tube. The LED strips were powered by a power bank and switched on via a light sensor^52^. Sampling of UV traps (*n* = 4) took place from 2022 to 2024 during June, July, and August (19 nights = 76 replicates). This data was used to determine intervals of peak attraction to ALAN and to set a temporally consistent baseline for the local light sensitive insect community. Road lights were sampled from 2023 to 2024 (13 nights = 78 replicates). During the two overlapping years, road lights and UV traps were sampled simultaneously on the same nights (Supplementary Table S10). Each sampling period consisted of two to three consecutive nights. Sampling periods were chosen to coincide with the waxing moon phase. Mean sunset time during summer from June to August was at 09.12 pm (min 20.10 pm, max 09.36 pm). The mean nightly temperature in the study area during the sampling period was 16.0 °C and ranged from lowest (9.3 °C) on 25.08.2024 to 22.9 °C (2022) on 20.07.2022 (data DWD, station ID: 03509). To capture variation in insect attraction throughout the night, each sampling night was divided into five-time intervals**:** interval 1 “early evening” (07:30 pm–09:30 pm), interval 2 “late evening” (09:30 pm–11:30 pm), interval 3 “midnight” (11:30 pm–01:30am), interval 4 “pre-dawn” (01:30am–03:30), interval 5 “dawn” (03:30am–6:00am). These intervals were defined to include both crepuscular and fully nocturnal samples.

Interval 1, interval 2 and interval 5 periods combine twilight conditions and crepuscular species, while interval 3–4 are expected to consist of exclusively nocturnal species. The replacement of collection containers took ~30 min per round, ensuring continuous data collection without prolonged exposure of samples. During each collection, insects were separated from the ethanol preservative using a sieve, after which the collection containers were refilled with the filtered fluid. The captured specimens were transferred into labelled ethanol-filled screw-cap jars, with labels specifying trap type, interval, date, and trap number. 100% ethanol preservation prevented specimen degradation, enabling accurate taxonomic identification and later analysis. Following sample collection, insects were identified to the order level using a microscope and counted. This was done to ensure consistency and feasibility across sampling efforts, working with various high school students. The sampling was conducted as part of a student-led project in which two to three high school students per year participated as citizen scientists. The students performed the fieldwork as part of their coursework, contributing to all stages of data collection and analysis. The results of their study were presented in “*Jugend forscht”* (https://www.jugend-forscht.de/information-in-english.html), a German youth science competition that promotes young researchers by encouraging independent scientific projects and fostering interest in STEM fields.

### Statistics and reproducibility

Statistical analyses were carried out with R-Studio Version 2024.04.2+764 and conducted separately for UV traps and road lights. Abundance (dependent variable) was calculated by counting individuals per taxon in each treatment, trap and interval. To consider variation between individual traps and sampling dates, we performed generalized linear mixed modelling with the “glmmTMB” package^66^. Model selection included distributions tests, AIC (Aikaike Likelihood criterion) and a backward selection of fixed effects. Hypothesis testing was carried out using the “anova” function of the “stats” package^67^. We constructed two separate models for the complete data sets of i) UV ground traps and ii) Road lights. The distribution family was set to “nbinom2” for both models.

i) Model selection was conducted using a stepwise comparison of candidate models, evaluated using Akaike’s Information Criterion (AIC), likelihood ratio tests and changes in log-likelihood. A model including only interval as a fixed effect (Model 6) had an AIC of 2911 (Supplementary Table S11). Adding year as a random effect improved model fit (Model 5, AIC = 2905). While inclusion of trap identity (Model 4) only produced a slight further reduction in AIC (2904), this random factor was kept in the model to account for repeated measurements and hierarchical sampling structure. The lowest AIC was obtained for the model additionally including an observation-level random effect (Model 3, AIC = 2898), indicating that this random-effects structure provided the best fit to the data. In a second step, environmental covariates were added to the selected random-effects structure. A model including nightly mean temperature (Model 2) further reduced the AIC to 2893. Inclusion of sunset timing (h) further improved model fit, resulting in the final model (Model 1) with interval, nightly mean temperature and sunset timing as fixed effects and the lowest AIC value (2878). Continuous predictors were mean-centred prior to final model fitting to improve interpretability and reduce collinearity. Simulated residuals were generated using 250 simulations (DHARMa package^68^) and residual plots were visually inspected. Formal diagnostic tests were then performed to assess residual uniformity, dispersion, zero inflation and normality of random effects. In addition, we assessed autocorrelation across both within-night intervals and across sampling dates using the “testTemporalAutocorrelation” function applied to simulated residuals. Durbin–Watson statistics indicated no significant temporal autocorrelation for any grouping variable. Residuals grouped by sampling date showed no evidence of autocorrelation (DW = 2.03, *p* = 0.94). Similarly, no significant autocorrelation was detected for sampling interval (DW = 2.24, *p* = 0.77), sunset timing (DW = 1.92, *p* = 0.85) or nightly mean temperature (DW = 1.84, *p* = 0.72). Collectively, these results suggest that residuals were temporally independent and that the final model adequately accounted for temporal structure in the data.

ii) Model selection was conducted using a stepwise comparison of seven candidate generalized linear mixed models (Supplementary Table S12). The simplest model containing only interval as a fixed effect (Model 7) showed the poorest fit (AIC = 1639). Sequential addition of random intercepts for year (Model 6, AIC = 1630) and site ID (Model 5, AIC = 1612) substantially improved model performance, indicating that temporal and spatial variation explained a large proportion of the observed variance in insect abundance. Inclusion of treatment as a fixed effect further improved fit (Model 4, AIC = 1602), while the interval*treatment interaction resulted in an additional reduction in AIC (Model 3, AIC = 1592), supporting differential treatment responses across sampling intervals. Adding nightly mean temperature significantly improved model fit relative to Model 2 (AIC = 1517). Similarly, inclusion of sunset timing did further provide additional explanatory power (Model 1, AIC = 1513). Although the coefficient for nightly mean temperature in the final model (Model 1) was negative, the variable was retained because temperature is a biologically important predictor of nocturnal insect activity and significantly improved model fit. In addition, analyses of the UV-trap dataset showed the expected positive relationship between temperature and insect abundance, supporting the ecological relevance of this covariate. Temperature was therefore retained to account for environmentally-driven variation among sampling nights and to prevent unexplained environmental variance from inflating or confounding estimates of the interval-treatment interaction effects. Residuals showed no evidence for temporal autocorrelation among intervals (DW = 1.1394, *p* = 1.1394), nightly mean temperatures (DW = 1.5864, *p* = 0.4381) or sunset timing (DW = 1.9561, *p* = 0.9351) after accounting for the model structure.

To evaluate and visualise predictor effects, estimated marginal means and model predictions were calculated using the R packages emmeans and ggeffects^69,70^. Predicted abundances and corresponding 95% confidence intervals were extracted for the categorical interval variable as well as for the continuous predictors nightly mean temperature and sunset timing. For the interval factor, pairwise post hoc comparisons among intervals and treatments were conducted using Tukey-adjusted estimated marginal means (H1, H2). For continuous predictors, model predictions were generated across evenly spaced gradients of nightly mean temperature and sunset timing. To improve interpretability in figures, centred predictor values were back-transformed to their original measurement scales using the corresponding means and standard deviations from the original dataset. Results were visualised using the R package ggplot2^71^. For interval effects, raw abundance observations were displayed as jittered points together with model predicted abundances and 95% confidence intervals (error bars). Significant differences among intervals were indicated by compact letter displays. For nightly mean temperature and sunset timing, predicted abundance responses were illustrated as fitted response curves with shaded 95% confidence intervals.

To provide a visual ordination and to evaluate the influence of individual insect taxa on compositional differences between intervals and treatments (H3), a Principal Component analysis was performed on the centred log-ratio (clr) transformed community matrix (order-level abundances aggregated per trap, light treatment and interval) with the “prcomp” function (scale = T), which was also conducted separately for UV traps and road lights. Data was preprocessed with the “multLN” function of the package “compositions” prior to clr transformation to replace zeros (z. warning: 0.7)^72^. A biplot was used to visualize results. In addition, we plotted the Eigenvalues in a scree plot to determine how many PCs to examine (threshold = 1/number of variables). To understand how each taxon is correlated, we considered taxa with a loading higher than the square of 1/number of variables in our interpretation of the principal components. To extend the visual interpretation with a statistical test for differences in the community composition between light treatments and intervals (groups) (H3), we performed Permutational Multivariate Analysis of Variances (PERMANOVA) with the “adonis2” function on the square root transformed community matrix (permutations = 999, method = “bray”, strata = Luminaire ID). This included treatment * interval as fixed effects in the analysis of road lights and only the interval for UV traps. Dispersion tests (“betadispers”) showed no significant differences in within group dispersion among intervals or treatments.

### Ethics statement

This study involved field sampling of free-living insects in Germany, resulting in the capture and mortality of individuals. No experimental manipulation, housing or invasive procedures were performed. In accordance with applicable German regulations, ethical approval was not required for the collection of non-protected invertebrates. Sampling was conducted under relevant local permissions where applicable.

### Reporting summary

Further information on research design is available in the Nature Portfolio Reporting Summary linked to this article.

## Supplementary information

Supplementary Information

Reporting Summary

## Data Availability

The source data is available at Figshare via 10.6084/m9.figshare.29436527^73^ and URL: https://figshare.com/s/5b1995409424bfadb550. Files: Lighttraps_long.csv: The source data underlying Figs. 2A, 5, Supplementary Fig. S2, Supplementary Table S1; Lighttraps_wide.csv: The source data underlying Fig. 2B–D, Supplementary Tables S3, S11; Roadlights_long.csv: The source data underlying Figs. 3, 5, Supplementary Fig. S2, Supplementary Table S2; Roadlights_wide.csv: The source data underlying Fig. 4, Supplementary Fig. S1, Supplementary Tables S4, S12; Principal_component_analysis_neuglobsow_matrix.csv: The source data underlying Fig. 6, Supplementary Tables S4–S8.

## Code availability

All code used in this study is available at Figshare via 10.6084/m9.figshare.29436527^73^ and URL: https://figshare.com/s/5b1995409424bfadb550. Files: R_script_intervals_MD_05-05-26.txt: R-Code used for data processing and statistical analyses. The analyses additionally used the original DWD Climate Data Centre dataset “produkt_tu_stunde_20050101_20241231_03509.txt”, comprising hourly air temperature observations used to calculate means in this study. These data are provided by DWD under the Creative Commons Attribution 4.0 International (CC BY 4.0) licence and are available from the DWD Climate Data Centre Open Data portal. URL: https://opendata.dwd.de/climate_environment/CDC/observations_germany/climate/hourly/air_temperature/historical/stundenwerte_TU_03509_20041001_20241231_hist.zip; accessed 24 April 2026
