# Supplementary material for: Citizen science reveals temporally structured insect attraction and mitigation efficacy under artificial light at night
Source: Commun Biol. 2026 Sep 21;9:1227. doi: 10.1038/s42003-026-10979-2 (PMC13594263; doi:10.1038/s42003-026-10979-2)
Supplement: Supplementary file 1 [file 42003_2026_10979_MOESM1_ESM.pdf]

Supplementary Information

Citizen science reveals temporally structured insect attraction and mitigation efficacy under artificial light at night

Corresponding Author: Manuel Dietenberger

Email: manuel.dietenberger@igb-berlin.de

Leibniz Institute of Freshwater Ecology and Inland Fisheries Berlin

Müggelseedamm 310

12587 Berlin

Table S1: Insect monitoring Neuglobsow UV ground traps 2022-204 (Overview). Number of attracted Taxa per light trap trap in Neuglobsow (Brandenburg, Germany). n=4, 19 nights, (x 5 intervals) from 2022-2024 (June to August).

Trap ID	Auchenorrhyncha	Brachycera	Coleoptera	Ephemeroptera	Hemiptera	Heteroptera	Hymenoptera	Lepidoptera	Megaloptera	Nematocera	Psocoptera	Sternorrhyncha	Thysanoptera	Trichoptera	abundance
1	96	128	571	78	82	52	107	1575	12	1143	17	15	43	471	4390
2	77	244	587	19	56	31	97	952	9	605	34	26	75	290	3102
3	30	77	503	42	49	48	103	552	10	521	7	6	14	232	2194
4	107	85	327	20	7	106	61	1343	9	685	17	10	10	355	3142
TOTAL	310	534	1988	159	194	237	368	4422	40	2954	75	57	142	1348	12828

Table S2: Insect monitoring Neuglobsow road lights 2023-204 (Overview). Number of attracted Taxa per treatment and trap in Neuglobsow (Brandenburg, Germany). Conventional HPS/ Shielded LED road lights, n=3 traps per treatment, 13 nights (x 5 intervals) from 2023-2024 (June to August).

Trap ID	treatment	Auchenorrhyncha	Brachycera	Coleoptera	Ephemeroptera	Hemiptera	Heteroptera	Hymenoptera	Lepidoptera	Nematocera	Psocoptera	Sternorrhyncha	Thysanoptera	Trichoptera	abundance
1	Shielded LED	0	14	43	28	27	1	6	3	57	2	0	19	4	204
2	Shielded LED	0	6	25	56	13	0	6	2	42	3	1	4	4	162
3	Shielded LED	1	12	25	11	13	0	11	3	61	3	0	3	2	145
4	Conventional HPS	2	24	44	12	27	14	20	16	279	1	0	8	21	468
5	Conventional HPS	0	12	46	75	20	3	16	6	172	2	0	8	9	369
6	Conventional HPS	1	16	27	114	31	8	18	8	126	1	0	5	6	361
	Total	4	84	210	296	131	26	77	38	737	12	1	47	46	1709

Table S3: Effects of time intervals on insect abundance at UV ground traps (generalized linear model). n=4 traps, 19 nights (x 5 intervals) from 2022-2024 (June to August). P-values are based on a two-sided Wald z-test. P-values lower than p=0.05 are printed in bold.

	**Estimate**	**Std. Error**	**Z-value**	**P-value**	
Interval 1 (Intercept)	2,39528899	0,20758899	11,5386128	8,43e-31	***
Interval 2	1,5592635	0,27944835	5,57979132	2,41e-08	***
Interval 3	1,27184756	0,27821697	4,57142335	4,84e-06	***
Interval 4	0,77836051	0,2789674	2,79014866	0,00526838	**
Interval 5	0,01572583	0,28304253	0,05555995	0,95569237	
Nightly mean temperature	0,11194315	0,02535	4,41590314	1,01e-05	***
Sunset timing (h)	-0,8103919	0,20106242	-4,0305489	5,56e-05	***

Table S4: Effects of light treatments and time intervals on insect abundance at road lights (generalized linear model). n=3 traps per treatment (Conventional HPS, Tailored and Shielded LED, 13 nights (x 5 intervals) from 2023-2024 (June to August). P-values are based on a two-sided Wald z-test. P-values lower than p=0.05 are printed in bold.

**Abundance ~**	**Estimate**	**Std. Error**	**Z value**	**P value**	
(Intercept)	0,97955533	0,16843691	5,81556215	**6,04e-09**	***
interval2	1,33568026	0,20787697	6,42534013	**1,32e-10**	***
interval3	1,41470452	0,20964845	6,74798452	**1,50e-11**	***
interval4	0,67011165	0,2158464	3,10457639	**0,00190552**	**
interval5	0,29653393	0,22979814	1,29041051	0,19690817	
Treatment Shielded-roadlight	0,32535017	0,23412765	1,38962727	0,16464209	
Nightly mean temperature	-0,1500821	0,02017628	-7,4385433	**1,02e-13**	***
Sunset timing (h)	-0,2383144	0,0961941	-2,4774329	**0,01323313**	*
Interval 2:treatment Shielded-roadlight	-1,3374652	0,30934138	-4,3235898	**1,54e-05**	***
Interval 3:treatment3 hielded-roadlight	-1,2805958	0,29883978	-4,2852254	**1,83e-05**	***
Interval 4:treatment Shielded-roadlight	-0,9694695	0,32362445	-2,995662	**0,0027385**	**
Interval 5:treatment Shielded-roadlight	-0,7267354	0,3448668	-2,107293	**0,03509219**	*

Table S5: Principal component analysis Neuglobsow UV traps 2022-204. Values depict loadings of individual insect taxa on Principal components 1-4. Loadings higher than 0.27 were considered important (bold numbers). Data based on. n=4 traps, 19 nights (x 5 intervals) from 2022-2024 (June to August).

	PC1	PC2	PC3	PC4
Auchenorrhyncha	**0,41064586**	-0,0601737	-0,146254	0,11529111
Brachycera	0,25998928	**0,35222684**	0,09124681	0,09453573
Coleoptera.	**-0,41037**	-0,0663656	0,01511125	0,0526143
Ephemeroptera	-0,2264375	**-0,3307149**	0,08827769	-0,0539356
Hemiptera	-0,1299624	**0,34907193**	-0,0099091	-0,569515
Heteroptera	0,24301177	-0,2524082	-0,3041482	0,10911751
Hymenoptera	0,06880887	**0,41835011**	0,06088311	-0,2606779
Lepidoptera	**0,32368853**	-0,220972	0,20551081	-0,2319473
Megaloptera	-0,2252182	-0,0791145	-0,5784699	-0,0614776
Nematocera	**0,38846094**	-0,1384482	0,00037418	-0,1724447
Psocoptera	0,13690447	0,26009187	0,2582187	0,58147032
Sternorrhyncha	-0,0423626	**0,27194866**	-0,5495991	0,29692826
Thysanoptera	**-0,3678339**	-0,0189714	0,34340165	0,23104217
Trichoptera	0,03434914	**-0,4263113**	0,04927862	0,03026333

Table S6: Principal component analysis Neuglobsow road lights 2023-204. Values depict loadings of individual insect taxa on Principal components 1-4. Loadings higher than 0.26 were considered important (bold numbers). Data based on n=3 traps per treatment (Conventional HPS, Tailored and Shielded LED, 13 nights (x 5 intervals) from 2023-2024 (June to August).

	PC1	PC2	PC3	PC4
Brachycera	0,11304651	0,06857456	-0,5213458	0,58698972
Coleoptera.	**-0,4337374**	0,25063453	0,13462442	-0,0720277
Ephemeroptera	0,22281594	**0,5434759**	0,21545844	0,0331361
Hemiptera	0,12406375	0,14134783	-0,3379886	-0,7528805
Hymenoptera	-0,0614251	-0,1386771	0,65597314	0,10296003
Lepidoptera	**0,2712641**	**-0,43706**	0,14238681	0,08058213
Nematocera	**0,50093833**	-0,1495495	-0,0590714	0,01250161
Psocoptera	**-0,4100819**	**-0,3656864**	-0,0219397	-0,1227159
Thysanoptera	**-0,4818669**	-0,0700191	-0,2920127	0,1481583
Trichoptera	0,07344568	**-0,4958705**	-0,097976	-0,1675333

Table S7: PERMANOVA results road lights. Differences in insect community composition between light treatments and time intervals. The results of this two-way PERMANOVA with interaction term (999 permutations, Bray-Curtis dissimilarity, stratified by luminaire ID) indicate significant variation among groups. Data based on n=3 traps per treatment (Conventional HPS, Tailored and Shielded LED, 13 nights (x 5 intervals) from 2023-2024 (June to August).

	**Df**	**SumOfSqs**	**R2**	**F value**	**P value**	
Interval	4	0,98820162	0,3759373	5,08360576	**0,001**	***
Treatment	1	0,32794074	0,12475709	6,74810232	**0,001**	***
Interval:Treatment	4	0,34054226	0,12955103	1,75185158	**0,019**	*
Residual	20	0,97194951	0,36975458			
Total	29	2,62863412	1			

Table S8: PERMANOVA results UV traps. Differences in insect community composition between light treatments and time intervals. The results of this two-way PERMANOVA (999 permutations, Bray-Curtis dissimilarity, stratified by luminaire ID) indicate significant variation among groups. Data based on. n=4 traps, 19 nights (x 5 intervals) from 2022-2024 (June to August).

	Df	SumOfSqs	R2	F value	P value	
Interval	4	0,89200959	0,76125225	11,9569543	**0,001**	***
Residual	15	0,27975652	0,23874775			
Total	19	1,17176611	1			

Table S9: Characteristics of light treatments. Photometric measurements of the light situations (mobiLux USB lux meter, Czibula & Grundmann GmbH Berlin, Jeti specbos 1201-UV spectrometer, Jeti Technische Instrumente GmbH Jena). Illuminance measurements were taken at four fixed vertical distances (0.75m, 2.25m, 3.75m, and 5.25m) across a range of increasing horizontal distance values (0.38m to 4.13m).

	**UV traps**	**Conventional HPS**	**Tailored & Shielded LED**
**Coordinates**	49.021079689. 08.377794075	49.411850863. 08.519436153	48.36368351 08.48160923
**Habitat**	grassland in resident gardens	riparian vegetation	riparian vegetation
**Traffic usage**	No traffic	Residential street <30km/h. Motorized vehicles + sidewalk	Residential street <30km/h. Motorized vehicles + sidewalk
**Light regime**	rural	rural	rural
**Luminaire Height (m)**	0.1	3.3	3.3
**No. luminaires per treatment**	4	3	3
**Sample events**	19 nights (2022-2024)	13 nights (2023-2024)	13 nights (2023-2024)
**Period**	June – August	June - August	June - August
**Illuminant**	50-cm-long 2835 SMD-LED-strip	HPS	LED
**Mean road Illuminance (lux)**	-	4.0	4.1
**Min road Illuminance (lux)**	-	0.9	0.3
**Spectral characteristics**	peak emission: around 400 nm	CCT (K): 2000	CCT (K): 2200

Table S10: List of Sampling dates in Neuglobsow. Mean nightly temperatures were derived from hourly data (09.pm-06.00am) from the closest nearby station of the German Wheather Forecast DWD (Station ID. 03509).

Roadlights	UV ground traps	Date	Date2	Nightly mean Temperature C	SUNSET	SUNRISE
	220624	220624	24.06.22	19,03	(21:40:04)	(04:42:42)
	220625	220625	25.06.22	19,38	(21:40:06)	(04:43:05)
	220719	220719	19.07.22	21,82	(21:24:27)	(05:05:52)
	220720	220720	20.07.22	22,85	(21:23:12)	(05:07:16)
	220819	220819	19.08.22	18,52	(20:29:40)	(05:56:12)
	220820	220820	20.08.22	15,51	(20:27:30)	(05:57:56)
230706	230706	230706	06.07.23	12,33	(21:36:49)	(04:50:17)
230707	230707	230707	07.07.23	14,98	(21:36:12)	(04:51:14)
230708	230708	230708	08.07.23	16,08	(21:35:31)	(04:52:13)
230709	230709	230709	09.07.23	18,12	(21:34:47)	(04:53:15)
240601	240601	240601	01.06.24	16,61	(21:25:32)	(04:48:43)
240602	240602	240602	02.06.24	13,66	(21:26:41)	(04:47:52)
240603	240603	240603	03.06.24	11,83	(21:27:47)	(04:47:05)
240714	240714	240714	14.07.24	15,1	(21:29:37)	(04:59:53)
240715	240715	240715	15.07.24	17,35	(21:28:33)	(05:01:09)
240716	240716	240716	16.07.24	14,56	(21:27:27)	(05:02:27)
240825	240825	240825	25.08.24	9,27	(20:15:18)	(06:07:22)
240826	240826	240826	26.08.24	11,07	(20:13:01)	(06:09:06)
240827	240827	240827	27.08.24	16,16	(20:10:44)	(06:10:49)

Table S11: Model selection UV ground traps.

Comparison of generalized linear mixed models with negative binomial error distribution for abundance data. Data based on. n=4 traps, 19 nights (x 5 intervals) from 2022-2024 (June to August). Models were compared using likelihood ratio test (χ^2 test), and model fit improved with increasing complexity, as indicated by decreasing AIC values. Model 1 showed the best fit. Fixed effects included time interval, nightly mean temperature and sunset time. Year and trap ID were included as random intercepts. P-values are based on a two-sided Wald z-test.

	**Df**	**AIC**	**BIC**	**logLik**	**deviance**	**Chisq**	**Chi Df**	**P value**
Mod 6	6	2911,39726	2934,31775	-1449,6986	2899,39726			
Mod 5	7	2904,56111	2931,30169	-1445,2806	2890,56111	8,83614717	1	**0,0030**
Mod 4	8	2903,6051	2934,16576	-1443,8025	2887,6051	2,95601157	1	**0,0856**
Mod 3	9	2897,58287	2931,96362	-1439,7914	2879,58287	8,02222527	1	**0,0046**
Mod 2	10	2892,60917	2930,81	-1436,3046	2872,60917	6,97369894	1	**0,0083**
Mod 1	11	2877,64855	2919,66946	-1427,8243	2855,64855	16,9606229	1	**3,82e-05**

Table S12: Model selection Road lights.

Comparison of generalized linear mixed models with negative binomial error distribution for abundance data. Data based on n=3 traps per treatment (Conventional HPS, Tailored and Shielded LED, 13 nights (x 5 intervals) from 2023-2024 (June to August). Models were compared using likelihood ratio tests, and model fit improved with increasing complexity, as indicated by decreasing AIC values. Model 1 showed the best fit. Fixed effects included time interval, treatment,their interaction, nightly mean temperature and sunset time Year and trap ID were included as random intercepts. P-values are based on a two-sided Wald z-test.

	Df	AIC	BIC	logLik	deviance	Chisq	Chi Df	P value
Mod 7	6	1638,72663	1660,80767	-813,36332	1626,72663			
Mod 6	7	1630,29211	1656,05331	-808,14605	1616,29211	10,4345287	1	**0,0012**
Mod 5	8	1611,74977	1641,19115	-797,87488	1595,74977	20,5423361	1	**5,83e-06**
Mod 4	9	1601,6362	1634,75775	-791,8181	1583,6362	12,1135695	1	**0,0005**
Mod 3	13	1591,59699	1639,43923	-782,79849	1565,59699	18,0392128	4	**0,0012**
Mod 2	14	1516,79422	1568,31664	-744,39711	1488,79422	76,8027626	1	**1,89e-18**
Mod 1	15	1513,21688	1568,41947	-741,60844	1483,21688	5,57734533	1	**0,012**

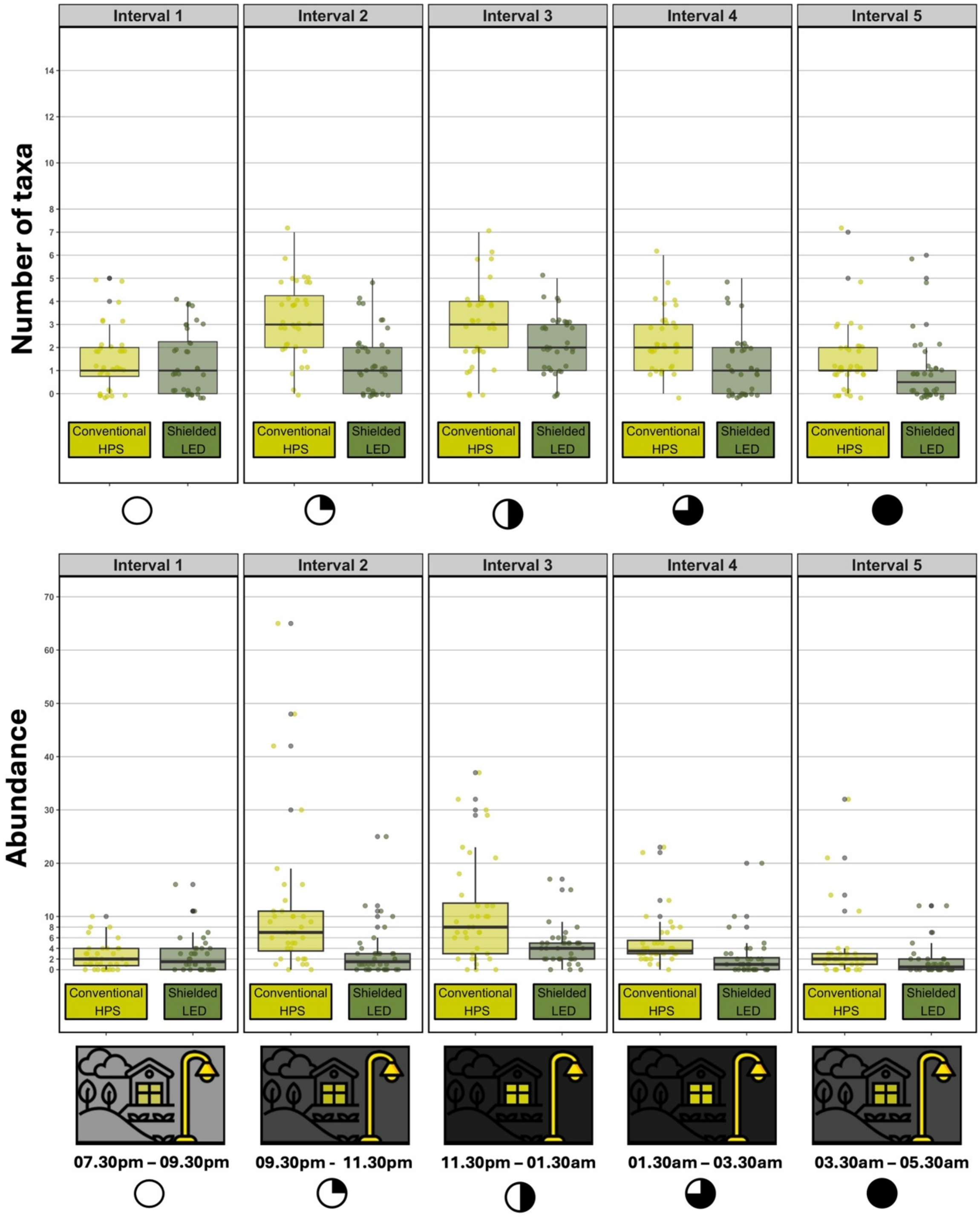

Figure S1: Number of taxa and abundance for time resolved road light samplings. Number of attracted taxa (upper panel) and individuals (Lower panel) (points) per treatment (x-axis) and time interval (facets). Conventional HPS vs. tailored and shielded LED road lights, n=3 traps per treatment, 13 nights (x 5 intervals) from 2023-2024 (June to August). Box plots

show median (thick line), interquartile range (IQR) (box), outliers (black points), 1.5 x IQR (whiskers). 667 sampling events in total (one trap in one night in one interval).

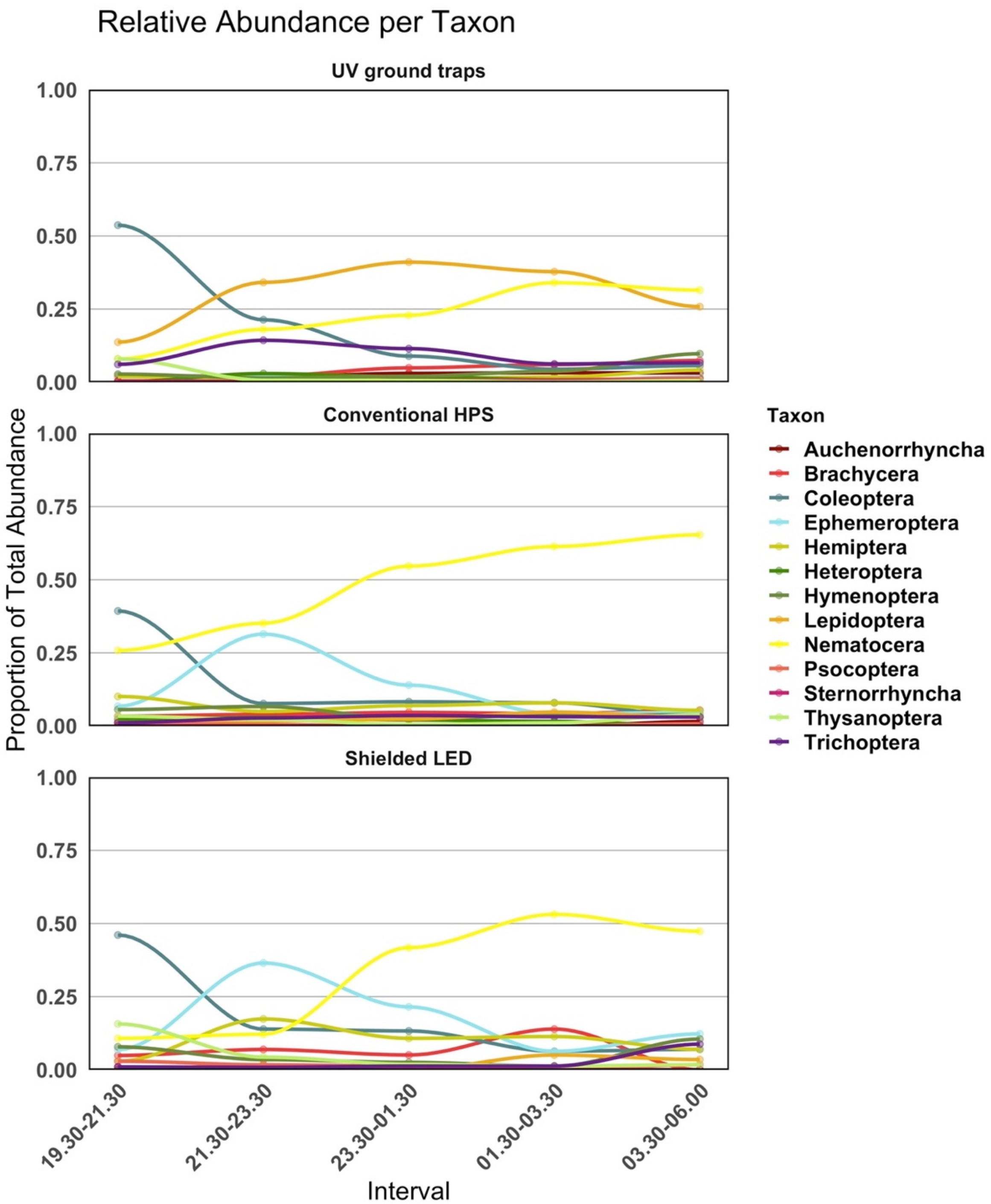

Figure S2: Proportion of Total abundances per road light treatment in different intervals. Smoothed temporal dynamics of relative insect abundance across taxonomic groups in different road light treatments. Each panel represents one treatment type (UV ground traps, conventional HPS roadlights, and shielded LED roadlights). Points indicate normalized taxon-specific proportions within each time interval, and LOESS-smoothed lines depict continuous changes

in relative abundance throughout the night. The y-axis shows the proportion of total nightly catch contributed by each taxon, allowing comparison of diel activity patterns between light sources.

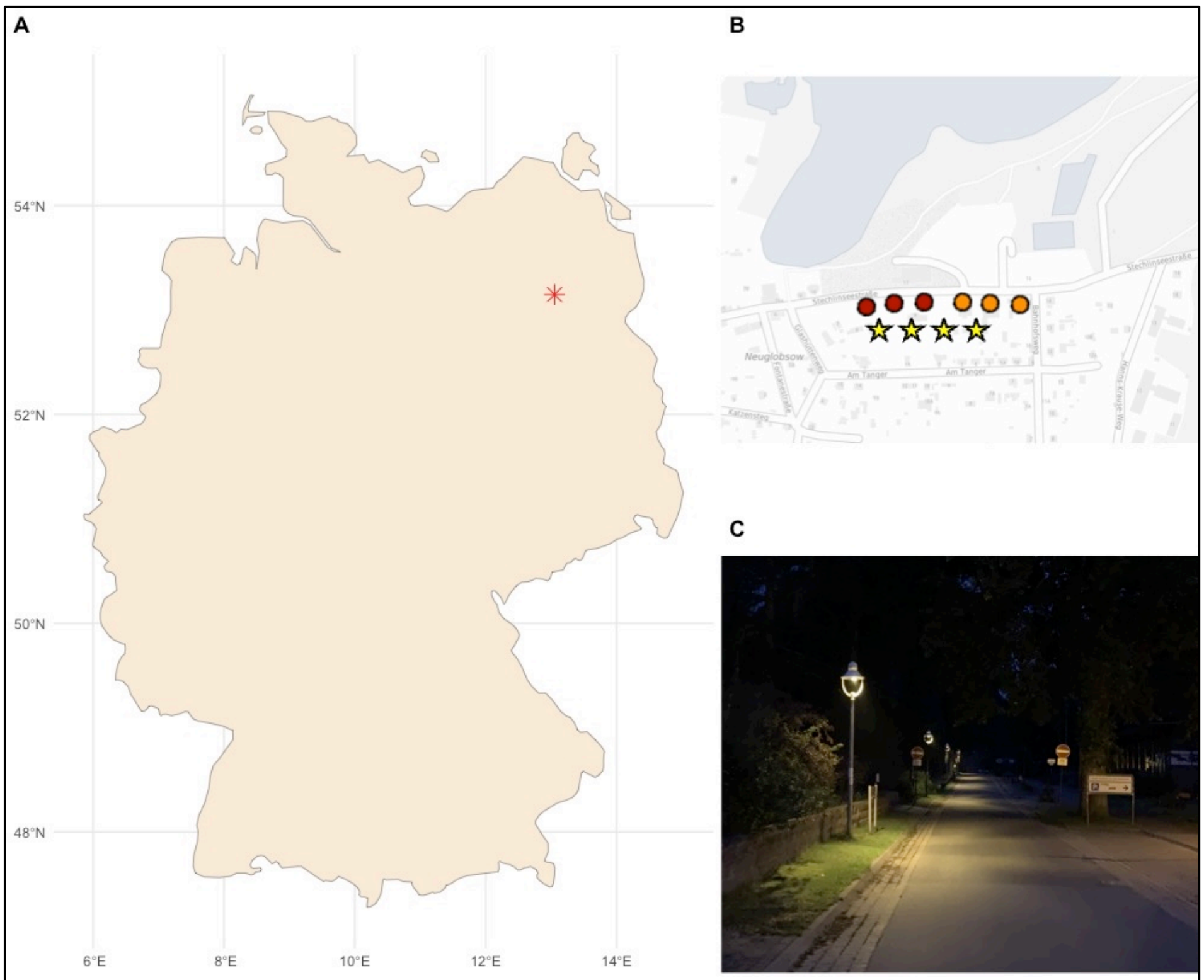

Figure: (A) Location of Neuglobsow in Germany. (B) Zoomed-in map of the study site. (C) Study site photograph.

Figure S3: Study site situated in North-eastern Germany (A) in Neuglobsow near lake Dagow. Traps at road lights are presented as circle. Red circles indicate the location of modernized tailored and shielded luminaires, orange circles conventional HPS). Stars represent the location of UV traps. UV traps were not visible from the road lights and vice verca. Photo by Johanna E. Reinhard. Maps data: Google, © 2024 CNES / Airbus, Maxar Technologies.

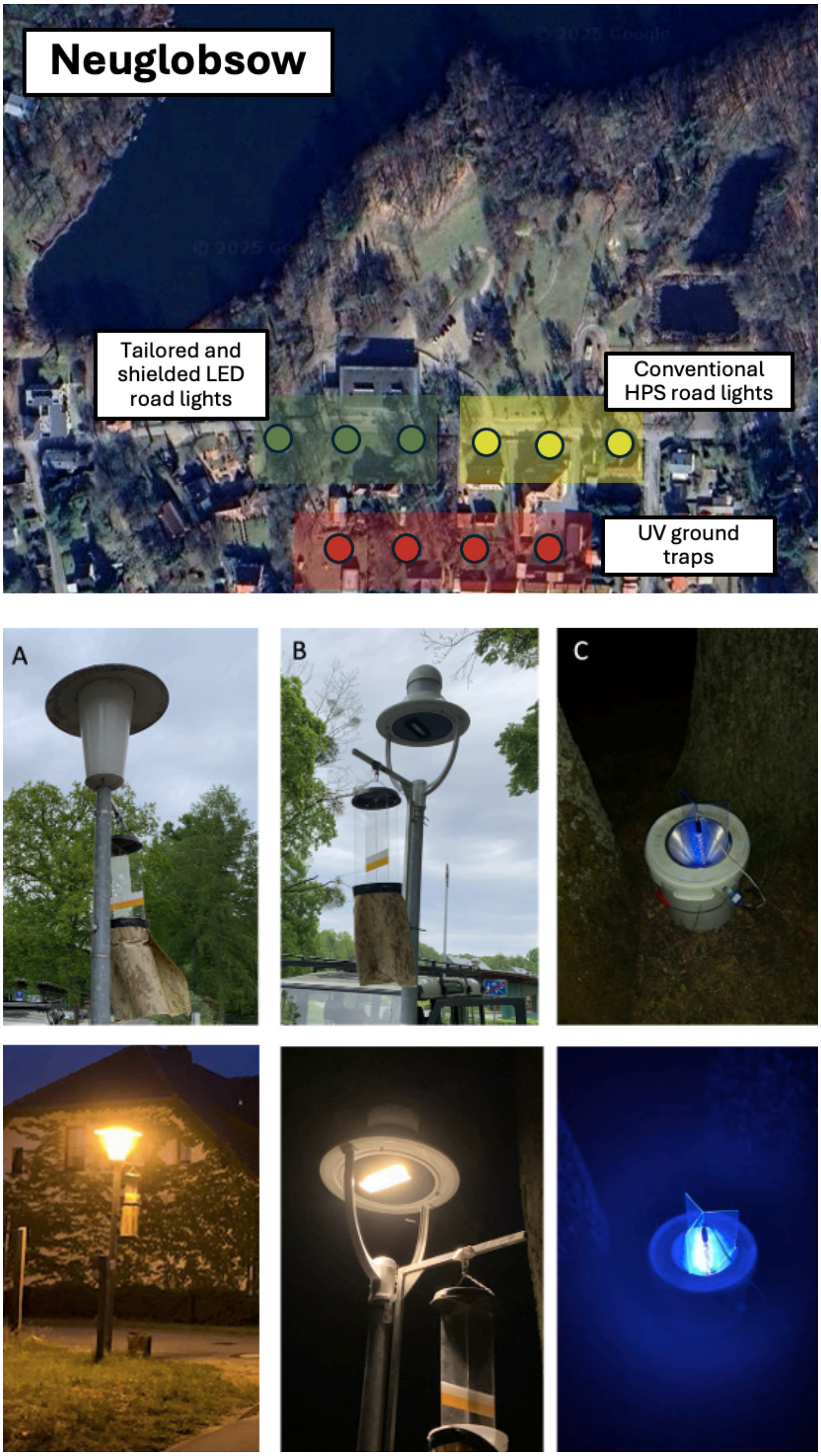

Figure S4: Luminaire and trap types. Top: Map shows locations of individual road lights/ UV traps. Bottom: HPS column luminaire with flight interception trap (3.3m height) (A), tailored and shielded luminaire with flight interception trap (3.3m height) (B) and UV traps on

ground level (C). Images taken in the first row during daytime in the second row during nighttime. Interception traps were installed at road lights using a conventional carrier for Christmas lighting. UV traps were placed within a bucket for life insect collections at the ground. Maps data: Google, © 2024 CNES / Airbus, Maxar Technologies. Photos by Johanna E. Reinhard / Andreas Jechow.

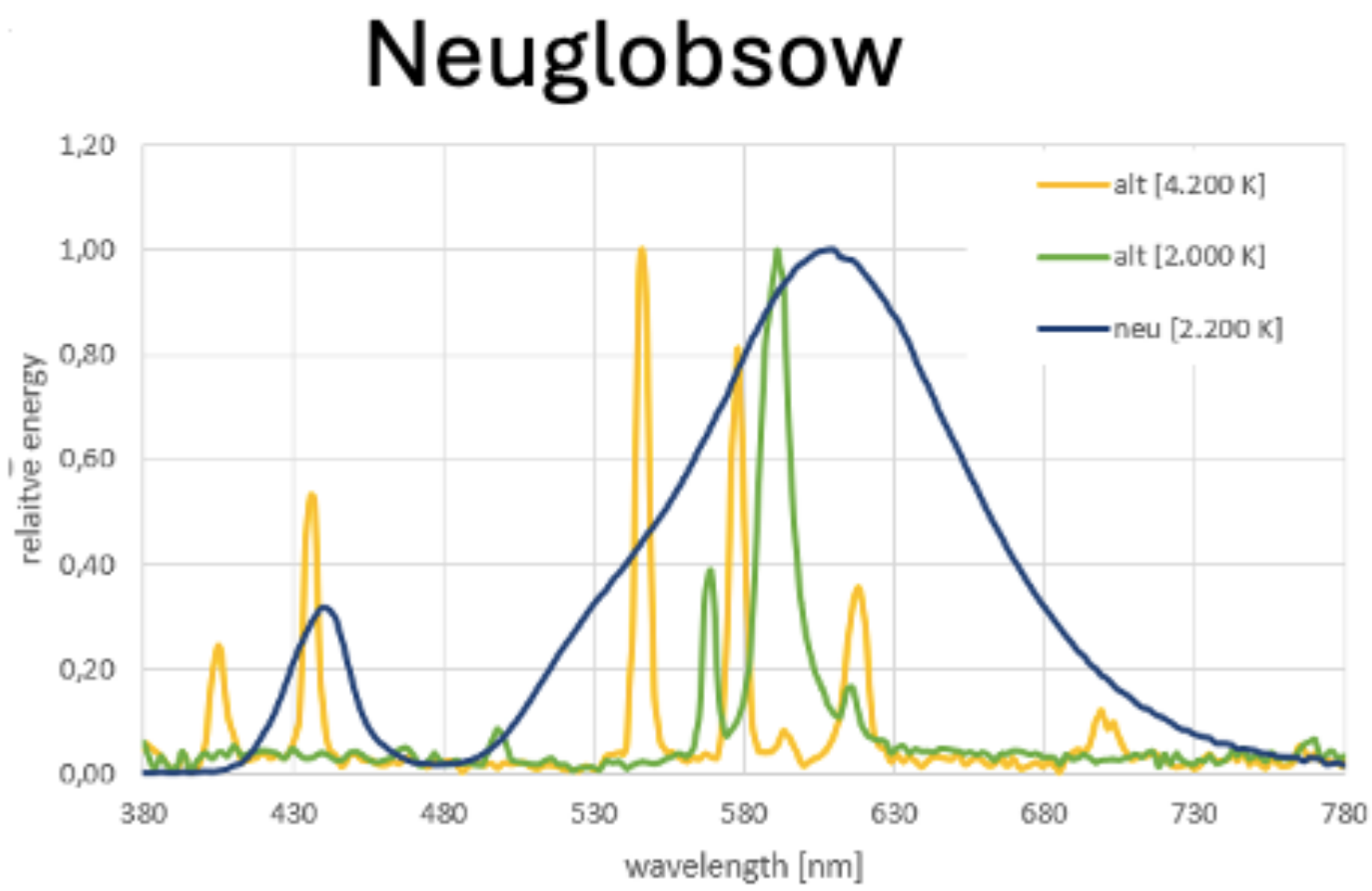

Figure S5: Individual spectra of road light treatments. The yellow curve shows the spectrum of luminaires installed before the experiment was conducted (MV luminaires, 0.98lx), not relevant. The green spectrum shows the conventional HPS control luminaires (4.0lx mean road illuminance), the blue line tailored and shielded LED luminaires (4.1lx). Photometric measurements of the light situations (mobiLux USB lux meter, Czibula & Grundmann GmbH Berlin, Jeti specbos 1201-UV spectrometer, Jeti Technische Instrumente GmbH Jena).
